# Computational Modelling of Metabolic Burden and Substrate Toxicity in *Escherichia coli* Carrying a Synthetic Metabolic Pathway

**DOI:** 10.3390/microorganisms7110553

**Published:** 2019-11-11

**Authors:** Martin Demko, Lukáš Chrást, Pavel Dvořák, Jiří Damborský, David Šafránek

**Affiliations:** 1 Systems Biology Laboratory, Faculty of Informatics, Masaryk University, Botanická 68a, 602 00 Brno, Czech Republic; 325073@mail.muni.cz; 2 Loschmidt Laboratories, Department of Experimental Biology and RECETOX, Faculty of Science, Masaryk University, Kamenice 5, Bld. A13, 625 00 Brno, Czech Republic; chr@mail.muni.cz; 3 Department of Experimental Biology, Faculty of Science, Masaryk University, Kamenice 5, Bld. A25, 625 00 Brno, Czech Republic; pdvorak@sci.muni.cz

**Keywords:** biodegradation, computational modelling, population growth, metabolic burden, environmental pollutants

## Abstract

In our previous work, we designed and implemented a synthetic metabolic pathway for 1,2,3-trichloropropane (TCP) biodegradation in *Escherichia coli*. Significant effects of metabolic burden and toxicity exacerbation were observed on single cell and population levels. Deeper understanding of mechanisms underlying these effects is extremely important for metabolic engineering of efficient microbial cell factories for biotechnological processes. In this paper, we present a novel mathematical model of the pathway. The model addresses for the first time the combined effects of toxicity exacerbation and metabolic burden in the context of bacterial population growth. The model is calibrated with respect to the real data obtained with our original synthetically modified *E. coli* strain. Using the model, we explore the dynamics of the population growth along with the outcome of the TCP biodegradation pathway considering the toxicity exacerbation and metabolic burden. On the methodological side, we introduce a unique computational workflow utilising algorithmic methods of computer science for the particular modelling problem.

## 1. Introduction

*Escherichia coli* strain BL21(DE3) is frequently used in synthetic biology with embedded protein expression (pET) vectors enabling heterologous protein expression [1,2,3,4,5,6]. Especially, it has recently found use as a cell factory for heterologous expression of entire biochemical pathways [7,8,9,10,11]. In spite of its common usage in metabolic engineering, the expression system in *E. coli* BL21(DE3) suffers from certain problems primarily caused by strong overexpression of recombinant proteins triggered by exposing the cognate ${\mathrm{LacI}}^{Q}/P_{lacUV5}$-T7 expression system to the synthetic inducer isopropyl-beta-D-thiogalactopyranoside (IPTG) [12]. Such negative effects that result from prioritising high levels of protein production to normal metabolic capacities in host cells are known as the *metabolic burden* [13,14]. One of the known factors causing the burden is the energetically expensive maintenance and replication of plasmid vectors carrying heterologous genes [15,16,17]. Other factors are associated with the activities of the foreign proteins which may interact with the metabolic network of the cell and burdens linked to the components of the expression system itself, such as IPTG. The individual factors may not always be additive, but can sometimes potentiate each other, leading to significantly larger effects on the living cells—the so-called *exacerbation effect*.

In our previous work, we designed and implemented a synthetic metabolic pathway for TCP biodegradation in *E. coli* [18]. The pathway was assembled with enzymes introduced to the cell using the pETDuet plasmids with ${\mathrm{LacI}}^{Q}/P_{lacUV5}$-T7 expression system inducible by IPTG. In relation to that work, we observed the effect of metabolic burden and toxicity exacerbation on *E. coli* population in [19]. However, the mechanisms behind these effects and their influence on cell growth have not been targeted. Deeper understanding of underlying mechanisms is extremely important for metabolic engineering of efficient microbial cell factories for biotechnological processes. To the best of our knowledge, the metabolic burden effect has not yet been fully-handled regarding the dynamics of affected metabolites and the influence on cell population growth. In [20], the authors reviewed existing fundamental frameworks for the kinetic modelling of microbial growth based on basic hypotheses about the underlying reaction systems. An integrated model presented in [21] couples the growth rate with the gene expression and the growth rate with the growing population of cells. To the best of our knowledge, existing population-level growth models neither consider metabolic interactions together with toxic effects caused by some metabolites nor describe a burden linked with using of engineered metabolic pathways. It is apparent that such effects cause a significant increase of the complexity of the underlying non-linear dynamics. To that end, a precise explanation of the resulting emergent behaviour remains a challenge.

**Figure 1:**
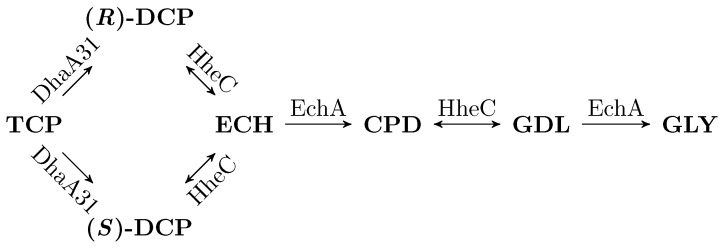
Model of metabolic pathway for biodegradation of TCP. A general scheme of an enzymatic metabolic pathway for biodegradation of TCP into GLY. Note that DhaA31 produces two different enantiomers of 2,3-dichloropropane-1-ol (DCP) with similar rate. However, enzyme HheC has notably different enantioselectivity with them. It is also worth noting that enzymes HheC and EchA are employed twice in the pathway. Other intermediates are epichlorohydrin (ECH), 3-chloropropane-1,2-diol (CPD), and glycidol (GDL).

The approach of computational systems biology gives a powerful tool allowing to study the dynamics of biological mechanisms holistically in terms of mathematical models. An important aspect of modern systems biology is the requirement to ground the models within the relevant genomic context of the explored organism [22]. It is obvious that utilising mathematical modelling and computational analysis based on the models makes an important starting point for successful experiment design in synthetic biology. To that end, the synthetic pathway we designed for conversion of the highly toxic TCP to glycerol (GLY) in *E. coli* [23] was optimised by using our original mathematical model [18] that allowed us to simulate the behaviour of the synthetic pathway in silico. The synthetic pathway is depicted in Figure 1. It is composed of a few toxic intermediates with harmless glycerol as a final product and it utilises enzymes from other bacterial species. These enzymes represent the engineered form of haloalkane dehalogenase (DhaA31, originally from *Rhodococcus rhodochrous* NCIMB 13064) and haloalcohol dehalogenase (HheC), and epoxide hydrolase (EchA) from *Agrobacterium radiobacter* AD1. They have a major role in this pathway; however, since they are heterologous proteins in *E. coli*, they have to be produced at the expense of other substances (causing a particular instance of the metabolic burden).

In this paper, we propose a significant extension of the existing mathematical model of the pathway which addresses for the first time the combined effects of toxicity exacerbation and metabolic burden in the context of bacterial population growth. To calibrate and fine-tune the model with respect to our original synthetically modified *E. coli* strain, we conducted several dedicated wet-lab experiments. Based on the new model calibrated with respect to the real data, we explore the dynamics of the population growth along with the outcome of the TCP biodegradation pathway considering all the phenomena mentioned above. On the methodological side, we introduce a unique computational workflow utilising algorithmic methods of computer science. It allows us to fully exploit the expressive power of the model under parameter uncertainty.

## 2. Materials and Methods

### 2.1. Laboratory Methods

#### 2.1.1. Bacterial Strains and Plasmids

*Escherichia coli* strain BL21(DE3) was used for this study with two modifications. Here, they are called deg31 (carrying synthetic metabolic pathway containing recombinant plasmids pCDF::*dhaA31* and pETDuet::*echA-hheC*) and host (carrying empty plasmids pCDF and pETDuet).

#### 2.1.2. Preparation of Pre-Induced Cells

The *lysogeny broth* medium (i.e., LB medium) with volume of 10 mL containing respective antibiotic combination (75 μg/mL ampicillin, 25 μg/mL streptomycin) was inoculated with transformed cells from glycerol stock. The culture was incubated overnight at 37 °C with shaking (180 rpm) in incubator Innova 44 (New Brunswick Scientific, Edison, NJ, USA—this machine was used for all incubations). Then, 25 mL of fresh LB medium with respective antibiotics were inoculated with 250 μL of night culture and incubated at 37 °C with shaking (180 rpm) until ${\mathrm{OD}}_{600}$ reached 1. The cultures were induced with 0, 0.01, 0.05, 0.2 and 1 mM IPTG and incubated overnight at 20 °C. Cells were then collected at late exponential phase by centrifugation (4000× *g*) at 4 °C in centrifuge Sigma 6-16K (Merck, Darmstadt, Germany—this machine was used for all centrifugation) and washed with 50 mM sodium phosphate buffer (NaP buffer, pH 7). After washing and centrifugation, cells were resuspended in NaP buffer to final value of 7 ${\mathrm{OD}}_{600}$.

#### 2.1.3. Short Term Toxicity Test

Prior to the toxicity test, 4 mM TCP was diluted in 5 mL of NaP buffer in 25 mL sterile Reacti Flasks closed by screw caps and incubated for 1 h at 37 °C. The reaction was initiated by mixing preincubated buffer with TCP with 5 mL of cells in NaP buffer, resulting in final ${\mathrm{OD}}_{600}$ 3.5 and 2 mM concentration of TCP. Reaction suspension was incubated in water bath at 37 °C with constant shaking for 5 h.

Cell samples were taken from the reaction mixture at the beginning of the toxicity test and after 4 h of incubation. Cell suspension (100 μL) was aseptically withdrawn from the flask and serially diluted in 900 μL of PBS buffer (pH 7.4) up to the final dilution of $10^{-6}$ to $10^{-7}$. Diluted cell suspensions (100 μL) was spread on Plate Count Agar (PCA) plates and incubated 24 h at 37 °C. After incubation, colonies on agar plates were manually counted and expressed as colony forming units per volume (CFU/mL).

#### 2.1.4. Growth Test

Cells of *E. coli* BL21(DE3) strain carrying empty plasmids and degrading strain deg31 were pre-grown in LB medium containing respective antibiotic combination (75 μg/mL ampicillin and 25 μg/mL streptomycin) at 37 °C until the culture reached stationary phase. The cells were then centrifuged at 6000× *g* and resuspended in Synthetic Mineral Medium (SMM) [18]. SMM medium (15 mL) with 10 mM glycerol in 25-mL Reacti Flask closed with screw cap was preincubated at 37 °C, growth test was initiated by addition of cells to 0.1 ${\mathrm{OD}}_{600}$. Growth medium was supplemented with IPTG to the final concentrations 0, 0.01, 0.05, 0.2 and 1 mM. Cells were incubated at 37 °C with shaking (200 rpm). Optical density (${\mathrm{OD}}_{600}$) of the culture was measured periodically and cell dry weight (CDW) in g/L at given time intervals was determined by multiplying the ${\mathrm{OD}}_{600}$ values by 0.39 g/L [24]. Acute toxicity measurement was performed in the same conditions, and the medium for toxicity test contained respective concentrations of TCP pathway metabolites.

#### 2.1.5. Glycerol Analysis

Samples withdrawn from incubated cell cultures were heated for 10 min at 95 °C, centrifuged and stored in the fridge prior to analysis. Free Glycerol Colorimetric/Fluorometric Assay kit (BioVision, USA) was used for analysis of glycerol content in the cells withdrawn from toxicity or growth tests following manufacturer’s instructions.

### 2.2. Parameter Constraints

We consider a set of *m* unknown parameters $p_{1},\ldots ,p_{m}$ and the set $P\subseteq R_{\geq 0}^{m}$ denoting the so-called *parameter space* consisting of *m*-dimensional vectors of parameter values. For the purpose of this paper, we assume P to be bounded. The constraints on parameters could generally be of various types. In this paper, we consider linear constraints of the form:

(1) $$a_{1}\cdot p_{1}+\cdots +a_{m}\cdot p_{m}∼b$$

where $p_{j}\in R_{\geq 0}:j\in \left\{1,\ldots ,m\right\}$ is a parameter; $a_{j},b\in R$ stand for coefficients; and ∼∈{<,>,≤,≥,=,≠} represents (in)equalities.

### 2.3. Biochemical Model

**Figure 2:**
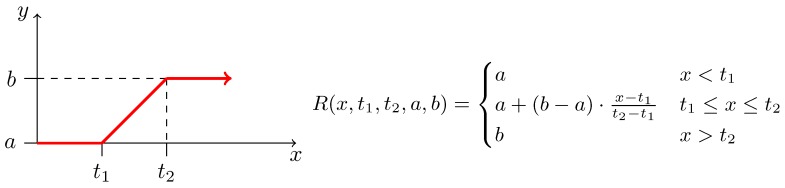
Definition of the ramp function. (**Right**) A mathematical definition of an increasing ramp function as used in our workflow. Parameters *a* and *b* are usually set to values 0 and 1, respectively; and vice versa for decreasing version. Values $t_{1}$ and $t_{2}$ typically represent some significant thresholds on *x*. (**Left**) Graphical description of the same function.

**Figure 3:**
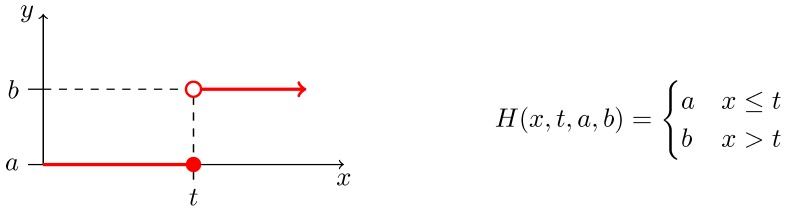
Definition of the Heaviside step function. (**Right**) A mathematical definition of an increasing Heaviside step function as used in our workflow. Parameters *a* and *b* are usually set to values 0 and 1, respectively; and vice versa for decreasing version. Value *t* typically represents an important threshold in the domain of *x*. (**Left**) Graphical description of the function—specifically, the increasing version.

A biochemical model, in this context, is a dynamical system given as a set of *ordinary differential equations* (ODEs) of the form

(2) $$\begin{array}{c} {\dot{x}}_{i}=f_{i}\left(\vec{x},\vec{p}\right):i\in \left\{1,\ldots ,n\right\} \end{array}$$

(3) $$\begin{array}{c} \vec{x}=\left(x_{1},\ldots ,x_{n}\right)\in R_{\geq 0}^{n} \end{array}$$

(4) $$\begin{array}{c} \vec{p}=\left(p_{1},\ldots ,p_{m}\right)\in P \end{array}$$

where $\vec{x}$ is a vector of *n* variables, $\vec{p}$ is a vector of *m* model parameters, and $f_{i}$ is a kinetic function constructed as a sum of reaction rates where every sum member represents an *affine* or *bi-linear* function of $x_{i}$; a *ramp* or a *Heaviside step* function of $x_{i}$ (Figure 2 and Figure 3, respectively); or a function from a limited set of *non-linear* functions of $x_{i}$ referred as sigmoidal functions; all of them possibly containing own set of internal parameters.

In general, our framework covers *mass action* kinetics ([25], Section 1.1) with stoichiometric coefficients not greater than one and all biologically relevant non-linear functions such as *Michaelis–Menten* [26] or *Hill* kinetics [27], and microbial growth kinetics such as *Monod* [28]. An additional requirement restricts the role of parameters in kinetic functions; in particular, we require $f_{i}$ is affine in $\vec{p}$.

### 2.4. Inverse Problem

An inverse problem in mathematics is a process of finding a good model (with parameters) reflecting given data; mathematically speaking:

(5) M(π)=d

where π is a vector of parameters of a model M and *d* represents the given data.

Intuitively, the problem is the following: “How to find the proper set of parameter values π given a model M.” In this paper, we distinguish two classes of the inverse problem—*parameter estimation* and *parameter synthesis*.

Parameter estimation is based on optimal fitting of the model parameters to observed experimental data. In the basic case, we assume the model to be a parameterised curve.

Parameter synthesis is a method that allows identification of satisfiable parameter values with respect to a given set of hypotheses restricting systems dynamics (described in a particular formalism) and a priori known parameter constraints (e.g., correlations of parameter values, constraints on production/degradation ratio, etc.). In this case, the parameters are assumed to be the model parameters (typically, the rate coefficients appearing in kinetic functions) [29,30,31,32,33,34,35,36,37,38,39,40].

#### 2.4.1. Parameter Estimation and Regression

Parameter estimation is a well-known process for the identification of the model parameters from experimental data representing the observations of the system. The particular problem of finding a parameterised curve (i.e., function) that approximately fits a set of data is referred to as *regression*. A function is classified as either linear or non-linear concerning affinity of the fitted parameters. Consequently, the problem is classified as the *linear regression* or the *non-linear regression* according to the function class. In general, the linear regression is easier but of limited effectiveness and it is preferred if we know the function. In this paper, almost all concerned functions (i.e., models) belong to the non-linear class. Hence, we assume only non-linear regression further referred to as regression or fitting (for simplicity) [41].

#### 2.4.2. Parameter Synthesis and Robustness Monitoring

Both approaches rely upon a *temporal logic* for specification of the behaviour properties of dynamical systems (Section 2.3). Although several different temporal logics have been developed, they can all be determined in the context of paths (or runs) of the system, i.e., the infinite sequences of states describing the system dynamics in consecutive time events. Such sequences are encoded in temporal logic *formulae* [42].

Parameter synthesis performs comprehensive exploration of the continuous parameter space including the space of initial conditions. It provides a *qualitative* answer to the question “which settings of a model satisfy a given set of temporal properties”. It is not as widely used as parameter estimation and therefore many of the developed tools are still in a prototype phase [29,30,31,32,33,34,35,36,37,38,39,40].

Robustness monitoring enables *quantitative* evaluation of the robustness of a model with respect to a temporal property under some perturbation of parameters (typically, reaction rate constants or initial conditions) [43,44,45,46,47,48].

### 2.5. Analysis Workflow

Mathematical models in synthetic biology are often represented in terms of the ODEs system. These models are quantitative and their functionality relies on particular parameter values. Parameter estimation is often the only solution to obtain proper parameter values that fit with experimental observations. In this section, we present our methodology step by step. The presented workflow is an extension of the workflow we introduced in [37].

#### 2.5.1. General Assumptions

There are several preliminary assumptions upon which the workflow is formulated. First, the model must be a metabolic pathway (i.e., a linked series of chemical reactions catalysed by enzymes [49]). Next, we consider several assumptions limiting the experimental settings of the studied system:

1. We assume the total inducer concentration to be constant in the time frame of our interest. An inducer is supposed to have a function of an input parameter, and it would be an inadequate parameter should it be adjusted spontaneously over time. Otherwise, the inducer degradation rate would be needed either found in literature or extracted from experimental data.
2. The workflow is limited to protease-deficient bacterial strains (e.g., *E. coli* BL21). In particular, we assume the total concentration of every enzyme affecting the studied pathway is constant in the time frame of our interest. Moreover, no influx of the enzymes is permitted as a consequence of time-limited induction phase where the proteosynthesis takes place [50,51]. Additional synthetic processes are considered negligible in a microbial population stressed enough by the massive expression of (heterologous) genes during induction.
3. There occurs a metabolic burden effect caused by the heterologous genes expression during the induction process and possibly by the presence of an inducer itself which in a combined way affects the bacterial growth rate.
4. Finally, we assume the bacterial population is in the stationary phase after the induction process is finished.

These assumptions limit the use of the proposed workflow to studies of the synthetic metabolic pathway models describing the metabolite dynamics in a time frame of a few hours. Moreover, the workflow targets the protease-deficient bacterial strains assuming some of the enzymes are products of expression of heterologous genes initiated by a non-metabolisable inducer during the induction process. It is worth noting that these significant assumptions help to keep the mathematical model computationally feasible in terms of the number of potentially unknown parameters (thus, minimising the risk of over-parameterisation). In particular, the degradation rates of enzymes can be neglected in such settings.

#### 2.5.2. Workflow Description

In the previous section, we define a metabolic pathway as a chain of chemical reactions with metabolites as the inputs and outputs of the reactions catalysed by enzymes. This form of the system is assumed to be the *input* to the workflow. The mathematical vector of concentrations of all model enzymes can be understood as the specific point in the *enzymatic space*.

In *Step 1* of the workflow, we focus on the reduction of the enzymatic space. Assuming that some of the enzymes are products of the induction process, their concentration can be expressed as the set of functions with an inducer as the input. There is one such function for each enzyme. The internal parameters of these functions need to be extracted out of experimental data—measured in various initial concentrations of the inducer—possibly with the help of parameter estimation methods. Typically, there are more enzymes than inducers in biochemical reactions. This step eliminates the extent of dependency of the model on enzymes and it provides the reduction of the number of potential model parameters.

Up to this point, the model was considered without any effect on the bacterial population. We propose to monitor and possibly predict an impact of the pathway on the population—such as the metabolic burden if using plasmids carrying heterologous genes—instead of modelling entire bacterial system. Following this idea, in *Step 2* of the workflow, we extend the current model with new variables describing: (1) the bacterial population; and (2) a substrate used to feed the population. In general, there can be more sources of nutrition. The dynamics of the extension is defined by the *growth rate* and *death rate* functions. Each rate function must depend at least on the particular substrate and might depend on the inducer and any of the metabolites especially if they have a harmful effect on the population. To extract a proper rate function, fitting of the internal parameters to experimental data is needed. The traditionally used growth functions include Monod, Moser, Tessier, etc. [52]. They can also be used for diauxic growth ([53], Section 12.1.3). At the cost of expanding the model with a few new variables, our framework allows predicting the population development depending on settings of the model parameters.

To fine-tune the model, we can use the formal computational methods of parameter synthesis mentioned in Section 2.4.2. This approach is more efficient than iterative analysis of demanding laboratory experiments used for fitting. Thus, in *Step 3* of the workflow, we need to specify a set of relevant parameters—ideally, the coefficients that can be altered in an experiment design allowing validation of the model-based results. Besides that, we need to formulate the temporal properties tailored to the particular case study. In general, this requires having some a priori knowledge about the investigated system. Nevertheless, there exist several typical (template) properties which can be used universally (i.e., oscillation, bistability, etc.). By using parameter synthesis together with robustness monitoring, we can: (i) specify various hypothetical scenarios where experimental data are missing or even impossible to achieve in laboratory conditions; (ii) monitor the behaviour of the investigated model; and (iii) optimise a set of tunable model parameters.

### 2.6. Software Tools

For the fitting procedure, we use several tools. GraphPad Prism (https://www.graphpad.com/) (version 8.2.1 (441)) is used in Section 3.3 to fit the functions describing the stable concentration level of enzymes induced by IPTG. The tool GUI is easy to use for traditional functions such as Michaelis–Menten. However, to investigate and compare the usability of a comprehensive set of functions, we use a more advanced tool—the FME package (https://cran.r-project.org/web/packages/FME/index.html) (version 1.3.5) [54] of the R language [55]. It offers well-documented procedures: parameter identifiability; parameter sensitivity; parameter fitting; and more.

For the parameter synthesis procedure, we utilise our tool Pithya [40], providing a parameter synthesis algorithm working with an expressive temporal logic ${\mathrm{HUCTL}}_{P}$ [39,56]. Its main advantage is it enables a fully automatised investigation of the system dynamics including exploration of equilibria. The tool has GUI and CLI, both available on github (https://github.com/sybila/pithya-gui) and also online (http://pithya.ics.muni.cz).

For the robustness monitoring, we use our tool Parasim that implements the algorithm working with a signal temporal logic ${\mathrm{STL}}^{*}$ [46,47,48] expressing properties of continuous signals. ${\mathrm{STL}}^{*}$ operates with forward consecutive time events over real-valued signals (i.e., evaluated variables). Its strength is to identify various types of repetitive behaviour (i.e., oscillations). The tool is available on github (https://github.com/sybila/parasim) and makes part of the online toolset BioDivine (http://pithya.ics.muni.cz/galaxy).

Both tools were successfully used on various biological case studies but so far they have been employed only separately (Pithya [34,37,38,56,57,58,59,60] and Parasim [46,48]). In this paper, we combine their application on a single model for the first time. Using the input files available online in shared history notebooks in the BioDivine framework, the results conducted in Section 3.5 with Parasim (http://pithya.ics.muni.cz/galaxy/u/martin/h/parasim-on-tcp-model-public) and Pithya (http://pithya.ics.muni.cz/galaxy/u/martin/h/pithya-on-tcp-model-public) can be reproduced.

## 3. Results and Discussion

In this section, we apply the sequence of steps defined in Section 2.5 to the model of TCP metabolic pathway (Figure 1).

**Figure 4:**
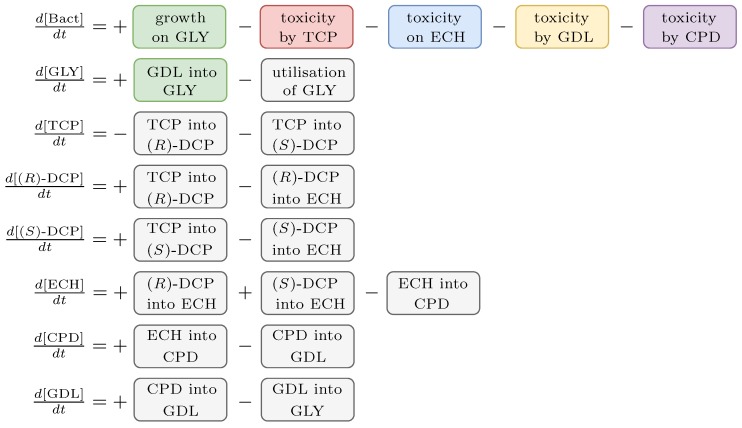
Proposed model scheme. A schematic description of the novel ODE model with highlighted extending partitions. Each partition (i.e., module) represents a linearly independent part of a particular equation. Each module has a unique semantic function. Note that some of the modules are used in more than one equation. The coloured modules represent entirely new parts of the final model and the grey modules were extended in this study. This modular feature was necessary to handle the fitting of such a complex model to the experimental data.

The outcome of the workflow is a novel model, the scheme of which is shown in Figure 4. It exhibits a modular structure, which was necessary to relieve the inverse modelling process (Section 2.4). We decided on this concept because the fitting of all new parts simultaneously would be practically impossible. Instead, only two to four parameters needed to be taken into account simultaneously. Due to this fact, the dedicated experimental data—capturing only partial behaviour—had to be obtained (Data S1–S3).

### 3.1. Extended Assumptions

Our model satisfies the general theoretical assumptions defined in Section 2.5.1: (1) the standard non-metabolisable IPTG inducer has been used [61]; (2) *E. coli* BL21 (DE3) is a protease deficient B strain, and all experiments were conducted in a closed environment with a limited amount of nutrients; (3) the core of the model is an expression of heterologous genes causing experimentally-provable metabolic burden [19]; and (4) all experiments were preceded by the induction phase after which the bacterial population was in stationary phase. Next, we assume the time frame of 5 h in which all metabolic pathway experiments and simulations were performed.

Next, we specify an extended set of assumptions characterising this case study:

1. We define a new variable called Bact standing for CDW (g/L) of *E. coli* population taken as 0.39 g/L = 1 ${\mathrm{OD}}_{600}$ [24].
2. We assume IPTG to be the only inducer for the synthetic pathway. Concentration of IPTG is considered to be constant in the given time frame.
3. Reversible reactions in the TCP-degradation pathway are considered negligible.
4. The initial concentration of substances (e.g., ${\mathrm{TCP}}_{0}$, ${\mathrm{GLY}}_{0}$, ${\mathrm{IPTG}}_{0}$) and the population (i.e., ${\mathrm{Bact}}_{0}$) determines the input for the system.
5. Dynamics of individual enzymes is approximated as a constant function of time in the given time frame. Moreover, enzymes dynamics is considered to be independent on the size of the bacterial population in the given time frame.
6. Total conversion of TCP into GLY is assumed to occur in a sufficiently long time reflecting the known behaviour of the pathway.
7. Viability of the bacterial population is given as the function of the pathway compounds toxicity, metabolic burden and the presence of nutrients (i.e., GLY).
8. Toxic effects of the pathway compounds are considered to be mutually independent.
9. Glycerol is the only assumed nutrient.
10. We assume natural degradation (death rate) of the bacterial population.

We are aware of the fact that Assumption (5) makes a significant approximation. The assumption reflects our former studies, in which we worked with pre-cultured pre-induced resting cells. In particular, estimated enzyme concentrations have been set as the endpoint values determined at a certain time interval after the overnight induction (Section 2.1.2).

### 3.2. Workflow Input: Synthetic TCP Degradation Pathway

Dvorak and co-workers [18] tested three different forms of the enzyme DhaA (two of them mutant) in order to improve the efficiency of the pathway. Nevertheless, we consider only the most effective form denoted DhaA31 (referred to here as DhaA for simplicity). This particular enzyme produces two different enantiomers of the DCP compound with a similar rate ($k_{\mathrm{cat},\mathrm{TCP},(R})-\mathrm{DCP}=0.58\;\left(s^{-1}\right)$ as the 55% of 1.05 ($s^{-1}$) for (*R*)-DCP and $k_{\mathrm{cat},\mathrm{TCP},(S})-\mathrm{DCP}=0.47\;\left(s^{-1}\right)$ as the 45% of 1.05 ($s^{-1}$) in favor of (*S*)-DCP). However, the enzyme HheC is enantioselective and prefers (*R*)-DCP ($k_{\mathrm{cat},(R})-\mathrm{DCP}=1.81\;\left(s^{-1}\right)$ vs. $k_{\mathrm{cat},(S})-\mathrm{DCP}=0.08\;\left(s^{-1}\right)$, where the greater means the better). As a consequence, (*S*)-DCP accumulates during the biodegradation process until (*R*)-DCP is consumed. Note that both enantiomers are supposed to be equally toxic to the host.

In general, reactions catalysed by HheC are reversible. However, in this particular case, the *catalytic efficiency* (i.e., $\frac{k_{\mathrm{cat}}}{K_{M}}$) of EchA in turning ECH into CPD is much higher than the catalytic efficiency of HheC towards the reaction ECH→(R,S)−DCP. Due to this fact, the reverse reaction was removed from the mathematical model ([23], Chapter 2). HheC also catalyses the reaction CPD↔GDL. The reverse reaction is compensated by a special kind of competitive version of Michaelis–Menten equation in the reaction GDL→GLY of the mathematical model. Thorough research can be found in [23]. However, we did a comparative test with several simulations of the model with competitive version of Michaelis–Menten from ([23], Chapter 2) and the simplified model containing only common Michaelis–Menten equations and, according to the results in Figure S1, we decided to use the simplified model instead (Figure S2).

### 3.3. Step 1: Enzymatic Space Settings and Reduction

**Figure 5:**
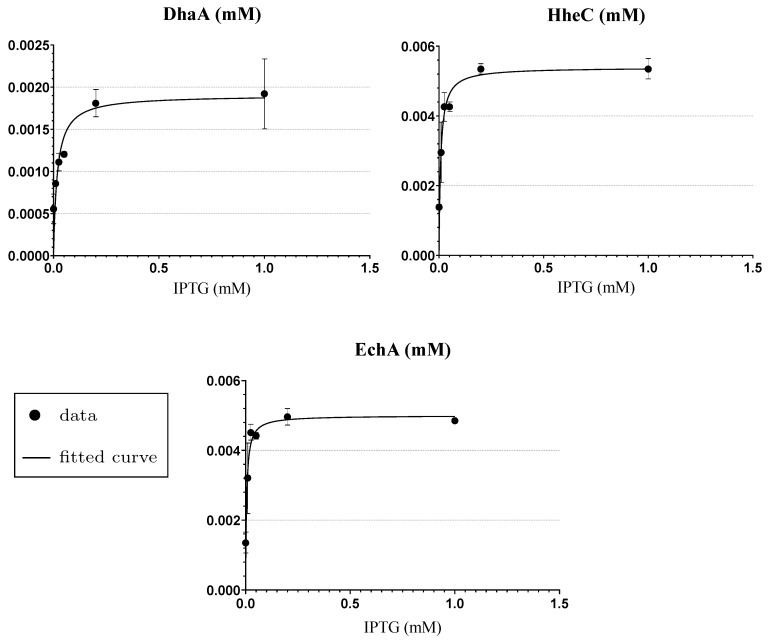
Results of fitting to enzymes concentration data. Three plots show individual results of fitting to concentration data measured in various starting concentration levels of the inducer (${\mathrm{IPTG}}_{0}$)—0.0, 0.01, 0.025, 0.05, 0.2 and 1.0 (mM)—for three different enzymes: DhaA (**top left**); HheC (**top right**); and EchA (**bottom**). The experimental data are pictured as points and the results—fitted curves—are pictured as lines. Both axes show a concentration level in mM, the inducer on the x-axis and the particular enzyme on the y-axis of the particular plot. Error bars represent standard deviation values calculated from two independent experiments.

Based on the simplifying assumptions mentioned above, the concentration levels of DhaA, HheC and EchA are represented as constants in the input mathematical model (Figure S2). In such settings, these constants can be understood as parameters. To extend the model with respect to IPTG concentration and in agreement with *Step 1* in Section 2.5.2, it is necessary to define functions describing the concentrations of enzymes depending on the concentration of IPTG. We extracted these functions by parameter fitting to experimental data obtained from densitometric analysis of enzyme cell-free extracts prepared from cells induced with different IPTG concentrations (more details in Appendix A). To that end, we employed Michaelis–Menten kinetics because the data showed a good agreement with its shape (Figure 5), although the MM is typically used for the description of a rate and not a concentration. The quantitative result of the fitting with statistical evaluation is shown in Table S1.

The initial growth of the functions is in perfect agreement with the *switch-like* influence when using a non-metabolisable inducer such as IPTG on ${\mathrm{LacI}}^{Q}/P_{lacUV5}$-T7 expression system [62,63] which is employed in heterologous plasmids containing genes of enzymes DhaA, HheC and EchA. The resulting functions of stable concentrations for particular enzymes are shown in Equations (6)–(8), respectively:

(6) $$\begin{array}{c} {\mathrm{DhaA}}_{total}=\frac{V_{\max,D}\;\cdot \;\left[\mathrm{IPTG}\right]}{K_{M,D}+\left[\mathrm{IPTG}\right]} \end{array}$$

(7) $$\begin{array}{c} {\mathrm{HheC}}_{total}=\frac{V_{\max,H}\;\cdot \;\left[\mathrm{IPTG}\right]}{K_{M,H}+\left[\mathrm{IPTG}\right]} \end{array}$$

(8) $$\begin{array}{c} {\mathrm{EchA}}_{total}=\frac{V_{\max,E}\;\cdot \;\left[\mathrm{IPTG}\right]}{K_{M,E}+\left[\mathrm{IPTG}\right]} \end{array}$$

with $V_{\max,D}=0.001904$, $V_{\max,H}=0.005391$, and $V_{\max,E}=0.004998$ being maximum rate constants $\left(s^{-1}\right)$ and $K_{M,D}=0.01749$, $K_{M,H}=0.008255$, and $K_{M,E}=0.004855$ being Michaelis constants (mM).

### 3.4. Step 2: Integration with Population Growth

**Figure 6:**
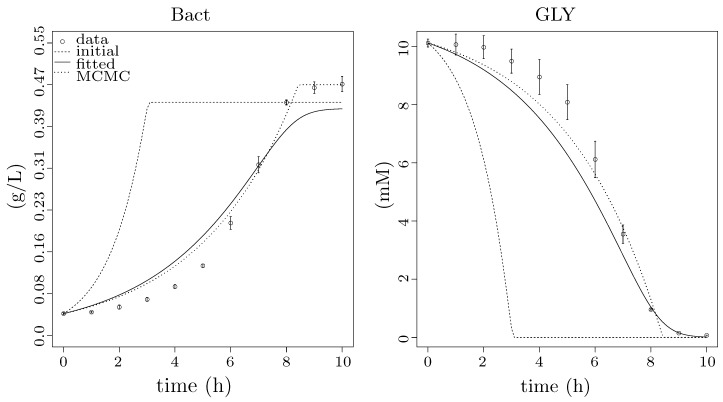
Results of fitting to population growth data. Two plots showing different results of fitting to population growth data from two points of view: (**left**) the particular results for CDW starting at 0.0429 and ending at 0.468 g/L after 10 h of growth; and (**right**) the result for the substrate utilisation only starting at 10.12 and ending at 0.07 mM after 10 h of growth. The experimental data are pictured as points with standard error bars, the dashed lines show simulation data for initial values of Monod function (i.e., initial point of fitting), the solid lines show the results of fitting (Section 2.4.1) and the dotted lines represent the final results optimised by MCMC method of the FME package (Section 2.4.1), which show the best agreement with the experimental data. The x-axes show time of experiment in hours; the y-axis of the right plot shows the concentration of GLY in mM; and the y-axis of the left plot shows CDW in g/L of bacterial population (Bact).

The original model does not take into account the bacterial population. Therefore, in agreement with *Step 2* in Section 2.5.2, we introduce a mechanism explaining the dependency of the substrate (GLY) and the inducer (IPTG) on the population. The Monod equation is commonly used to explain bacterial population growth rate μ, and, indeed, the results are in good agreement with experimental data (Figure 6). For the sake of completeness, we have exemplified several different alternatives (Tessier ([64], in French), Moser [65], Aiba–Edwards [66], Andrews [67], etc.). For the full list of considered functions, see the comparative table (Table S2). Nevertheless, according to Figure S3, the best results were observed by Monod growth model defined in Equations (9) and (10), where μ is the specific growth rate, $\mu_{max}$ is the maximum specific growth rate, *K* is the half-velocity constant, $\gamma_{GLY}$ is the rate of substrate utilisation and *Y* is the yield coefficient.

(9) $$\begin{array}{c} \mu =\frac{\mu_{max}\;\cdot \;\left[\mathrm{GLY}\right]}{K+[\mathrm{GLY}]} \end{array}$$

(10) $$\begin{array}{c} \gamma_{\mathrm{GLY}}=\frac{-1\;\cdot \;\mu }{Y} \end{array}$$

**Figure 7:**
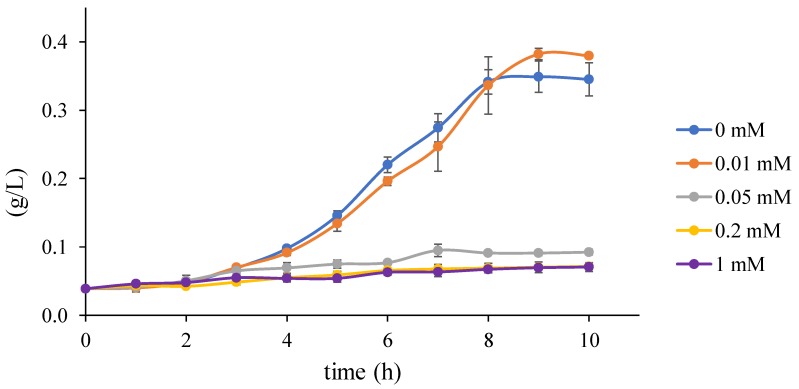
Bacterial population growth data reflecting concentration of inducer. The plot shows curves of population growth on GLY for cultures reflecting different concentrations of IPTG prepared according to Section 2.1.4. All cultures—carrying plasmids with heterologous metabolic pathway—started at the same value but ended with different size of population depending on the initial concentration of the inducer (${\mathrm{IPTG}}_{0}$): 0, 0.01, 0.05, 0.2, and 1 (mM). Note that the rising amount of IPTG led to progressive inhibition of bacterial growth. The most interesting is the big step from 0.01 to 0.05 of IPTG. This notable difference in the population on the relatively small interval of IPTG values and minimal changes in the population for the rest of IPTG concentrations shows the high sensitivity of the population to ${\mathrm{IPTG}}_{0}$.

**Figure 8:**
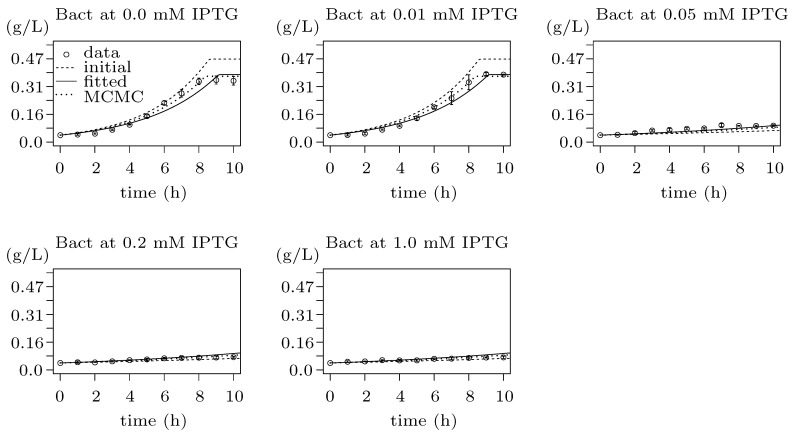
Results of fitting to population growth data reflecting metabolic burden caused by IPTG. The plot contains five figures, each showing fitting of the same model to the bacterial population growth data for different concentration of the inducer (IPTG) during 10 h long induction phase. The experimental data are pictured as points with standard error bars, the dashed lines show simulation data for initial values of the model function (i.e., initial point of fitting), the solid lines show the results of fitting and the dotted lines represent the final results optimised by MCMC method of the FME package (Section 2.6), which show the best agreement with the experimental data. The model with the best fit appears to be an enhanced Monod function where the maximum growth rate constant is substituted by the ramp function (defined in Figure 2) going from the maximum growth rate to the minimum growth rate reflecting the metabolic burden effect of the gradually-growing concentration of IPTG. The x-axes show the time of experiment in hours while the y-axes show CDW in g/L.

Traditional growth functions seem not to be sufficient to explain the effect of the metabolic burden caused by IPTG and heterologous genes expression, as displayed in Figure S4. Thus, considering the experimental data in Figure 7, we decided to use a specific function to model the gap between results for values $t_{1}=0.01$ and $t_{2}=0.05$ of mM ${\mathrm{IPTG}}_{0}$. It seems that the Heaviside step function (Figure 3) is a straightforward option for the apparent step in-between resulting values. However, as we do not know the exact value of the breaking point, it is safer to assume the linear behaviour. Hence, we decided to employ the ramp function defined in Figure 2 in the way that the maximum specific growth rate constant $\mu_{max}$—estimated above using Monod growth function (Figure 6)—is replaced with the ramp function defining an actual growth rate from the interval of the maximum ($\mu_{max}$) and the minimum specific growth rate ($\mu_{min}$) parameterised with ${\mathrm{IPTG}}_{0}$. The best result of the fitting is shown in Figure 8 and represents evidence of the metabolic burden caused by IPTG with heterologous genes expression.

For the completeness, we also assume a death rate coefficient ($\gamma_{\mathrm{Bact}}$), the value of which fits approximately in the range of [$3.876\;\times \;10^{-3}$, $5.382\;\times \;10^{-4}$] ($h^{-1}$). The uncertainty of these values comes from the fact that the death rate is usually not the main interest of microbiologists, and also some methods of measurement make the death phase hard to discern ([68], Section 3.1.4). The origin of the range values is explained in Appendix B and has the base in [69]. Although the range is an approximation, it is a good starting point for further investigation by parameter synthesis and robustness monitoring.

The decision of taking microbial population into account would not be complete without an understanding of the toxic effect of the pathway components. Here, the selection of the optimal function is not straightforward because no function has been made a standard for this purpose. Therefore, we investigated several functions (Appendix C). The best results are shown in Figures S5–S8 for TCP, ECH, CPD and GDL, respectively. Both DCP enantiomers were observed to have minimal effect on the population even for high doses (i.e., 4 mM).

With respect to the nature of the experimental data, some of the results were far from the best fit. Notably, the case of time-series data for TCP concentration of 2 mM was the worst (Figure S5). However, in the long time horizon, the population stabilises more or less on the same CDW (Figure S9). This fact indicates some delay in the cellular response to the presence of the toxic pollutant.

As we are interested in an explanation, or at least a mathematical description of the exacerbation effect, we introduced the Heaviside step function in the expression describing the TCP toxic effect on the bacterial population regarding of IPTG concentration. In other words, the purpose of the step function is to improve the TCP toxicity function according to an observation from the experimental data such that it simulates the exacerbation when TCP and IPTG effects are combined.

**Figure 9:**
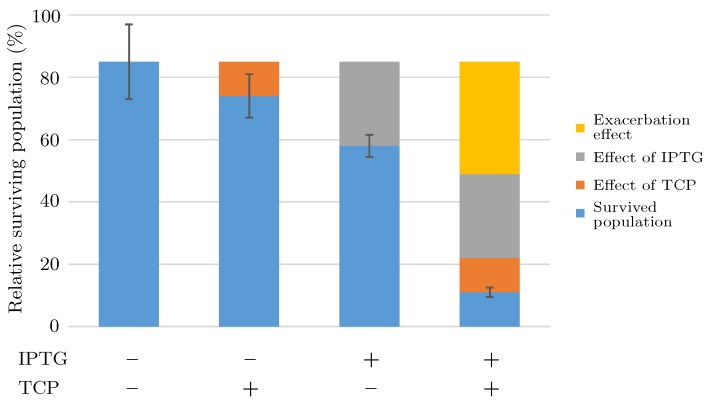
Evidence of exacerbation effect of IPTG on toxicity caused by TCP. Combined effect of metabolic burden caused by 0.2 mM IPTG and toxicity caused by TCP on *E. coli* BL21(DE3) cells carrying empty plasmids pCDF and pETDuet is displayed as the percentage of survived cells (blue bars) after induction in medium with or without 2 mM TCP. Pre-induced (IPTG +) or non-induced (IPTG −) cells were incubated in buffer with TCP (TCP +) or without (TCP −). The separate negative effects of TCP (orange), IPTG (gray), and the exacerbation of TCP toxicity in cells pre-induced with IPTG (yellow) are indicated. Error bars represent standard deviations calculated from at least five independent experiments. Note that experimental data come from [19].

In one case, a simple evidence of the exacerbation phenomenon has been given in [19] where Dvorak and co-workers compared the results of similar experiments of pre-induced (IPTG +) or non-induced (IPTG −) cells incubated in buffer with TCP (TCP +) or without (TCP −) (Figure 9 displays all four combinations: −−, −+, +−, and ++). The figure clearly shows some unexplained disruption amplifying the population extinction, although the data are not sufficiently convincing.

**Figure 10:**
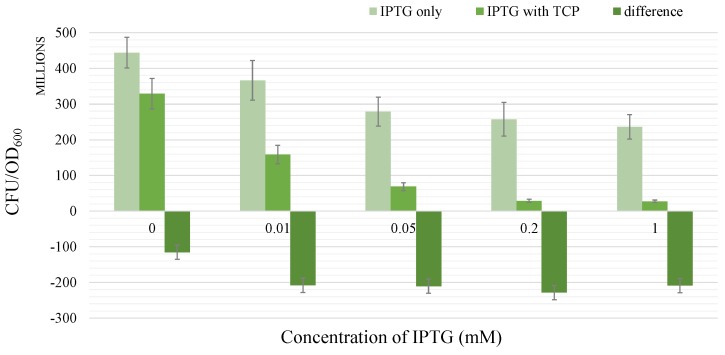
Exacerbation of TCP toxicity in *Escherichia coli* BL21(DE3) bearing synthetic TCP pathway by IPTG. The leftmost column in each dataset represents the population of cells pre-induced with various concentrations of IPTG. The middle column of each dataset shows the population of survived cells pre-induced with the same amount of IPTG after 5 h in the presence of 2 mM TCP. The rightmost column in each dataset shows the difference of the first and the second column (i.e., third=second−first). Note that the population in the first column of the first dataset is pre-induced with 0 mM IPTG and incubated in absence of TCP, which makes it a control group. The second column in the same dataset shows the sole effect of 2 mM TCP on the population. It is remarkable that the third columns in all other datasets seem to be in perfect match and approximately 1.82 times bigger than the same column in the first dataset. We explain this fact by the existence of the exacerbation effect. Different concentrations of IPTG were used for the induction of the TCP pathway expression from pCDF and pETDuet plasmids. Error bars represent standard deviations calculated from at least three independent experiments except for the rigthmost column in each dataset where error bars represent standard error of values in these columns.

In another, more extensive case, two datasets for the same type of experiment with *E. coli* population degrading over time are compared for various initial concentrations of IPTG—${\mathrm{IPTG}}_{0}$ (Figure 10). The first dataset represents the individual effect of IPTG and the second one reflects the combined activity of IPTG and TCP. Remarkable is the fact that the exacerbation is more or less conserved for various values of ${\mathrm{IPTG}}_{0}$ except for the case when ${\mathrm{IPTG}}_{0}$ equals zero (displaying the toxic effect of TCP only). By acquiring a proportion of the median of the differences for various positive concentrations of ${\mathrm{IPTG}}_{0}$ to the difference of the individual TCP effect (where ${\mathrm{IPTG}}_{0}$ equals zero) we have obtained a value of 1.82. Due to the fact it is dimensionless, it is suitable for a wide variety of models regardless of the units used for determination of the bacterial population. Therefore, we incorporate this value as a potential exacerbation parameter ($ex_{\mathrm{on}}=1.82$, whereas $ex_{\mathrm{off}}=1$) in the next step of our workflow.

### 3.5. Step 3: Model Dynamics Exploration

**Figure 11:**
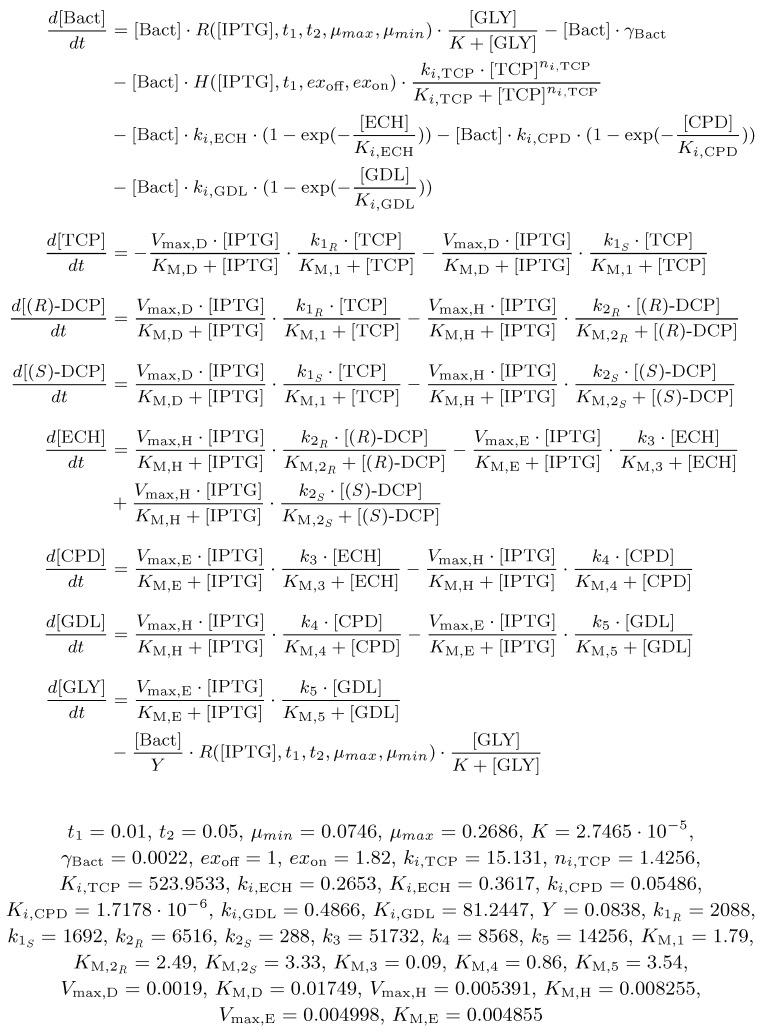
An ODE model of the extended TCP metabolic pathway. The model represents a chain reaction for biodegradation of TCP into GLY reflecting the *E. coli* population. Note that the rate constants of the original ODE model were rescaled from seconds ($s^{-1}$) into hours ($h^{-1}$) because the data used for fitting were sampled every hour. It concerns the original rate constants ($k_{1_{R}}$, $k_{1_{S}}$, $k_{2_{R}}$, $k_{2_{S}}$, $k_{3}$, $k_{4}$, $k_{5}$). Units: $k_{*},V_{*},\mu_{*},\gamma_{*}\left(h^{-1}\right)$; $t_{*},K_{*}\left(\mathrm{mM}\right)$; $ex_{*},Y,n_{*}\left(\mathrm{unitless}\right)$.

As the outcome of the previous part of the workflow, we obtained the extended ODE model (Figure 11). It makes an input into *Step 3* of the workflow (Section 2.5.2). For the purpose of the employed analysis techniques (parameter synthesis and robustness monitoring), we converted the model into two different formats compatible with the used software (the BIO (https://github.com/sybila/ode-generator/blob/master/README.md#model-syntax) format and the SBML (http://sbml.org/Documents/Specifications) format, respectively).

Next, we specify the model parameters that make the objectives of further analysis. While the previous parts of the workflow targeted the internal parameters adapting the model for the particular cell population, in this part, we target the model parameters that can be perturbed and fine-tuned. In particular, we consider the following set of model parameters for tuning:

1. **Concentration of IPTG in mM**: IPTG is an obvious candidate for tuning because many aspects of the model depend on it and it can be controlled easily in the experimental environment (since its concentration is considered constant during the experiment, it can be referred by its initial concentration, denoted ${\mathrm{IPTG}}_{0}$).
2. **Size of bacterial population (Bact) in g/L**: The initial population size, denoted ${\mathrm{Bact}}_{0}$, makes the crucial input of the model and it affects the model output—the final population size (reached in a given time). In general, the initial population size can be controlled in experiments.
3. **Initial concentration of TCP (**${\mathrm{TCP}}_{0}$**) in mM**: The key input of the model that must be set in order to make the modelled metabolic pathway work; it can be easily set to any arbitrary value during the experiments.
4. **Death rate of the population (**$\gamma_{\mathrm{Bact}}$**) in**$h^{-1}$: The death rate is considered as a parameter because we are interested in the dynamics of the microbial culture and the effects affecting the growth.

**Property** **1.** *The complete degradation of TCP as fast as possible with the least accumulated toxicity.*

In [37], we declared the desired property of the model dynamics verbally (stated as Property 1). The model used in that study did not concern the bacterial population. Therefore, the notion of toxicity was interpreted as an artificial accumulation of the inhibitory effect of the particular pathway’s (intermediate) products. The inhibitory effect was experimentally measured and is traceable in [18,23].

**Property** **2.** *The complete degradation of TCP as fast as possible with the most survived bacteria.*

In the extended model, we are able to investigate directly the effect of the particular pathway’s products on the bacterial population. To that end, the desired property is lifted to the population level resulting in Property 2. Due to the very abstract character, this property serves as a theoretical concept and several adjustments have to be performed to use it with computational analysis methods.

First, the part “*the complete degradation of TCP*” is translated into “*the TCP concentration is close to zero or below a minimal threshold (e.g., 0.01 of mM)*”. This is due to the possible errors of numerical solvers and the nature of the employed model approximation/abstraction algorithms.

Second, the terms such as “*as fast as possible*” and “*the most survived bacteria*” cannot be interpreted numerically, which is necessary for the computing. For that reason, we use various numerical thresholds to instantiate such abstract terms in the specified property.

As we addressed in Section 2.4.2, both considered approaches (parameter synthesis and robustness monitoring) employ a particular temporal logic for the specification of properties. We employ various properties compatible with both approaches and we utilise their specific advantages.

The results of parameter synthesis contain comprehensive information about parameter values for all possible initial settings of variables in considered ranges. In particular, not all *positive* results (parameter values satisfying the particular property) are automatically valid in all initial settings of model variables. The parameter synthesis method computes only positive results for which there exist some valid initial values of model variables. It is also worth noting that, due to the model overapproximation performed inside the parameter synthesis procedure, the complement to the set of positive results does not necessarily imply a valid negative result [37]. In other words, the so-called false-positives might exist.

**Property** **3.** *The population will not drop below 0.08 g/L until TCP will degrade fully (drop below 0.01 mM).*

**Figure 12:**
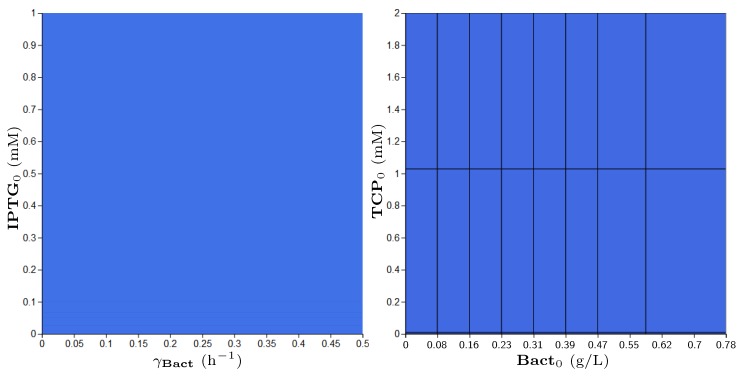
Results of parameter synthesis process for Property 3. Inside the figure, one can see two plots with blue regions. Both plots show a combination of parameters and (or) variables of the model where each point represents the particular evaluation of considered parameters (or variables). Every blue region represents a set of evaluations satisfying the stated property in at least one initial condition of the model. Here, the blue regions make joint projections across all non-displayed dimensions (i.e., parameters and variables). Consequently, the property holds in every combination of initial conditions (respectively, parameters) in the particular ranges.

Addressing Property 2, we designed a suitable reformulation (Property 3), which is compatible with the parameter synthesis procedure. The results of parameter synthesis for this property are shown in Figure 12. The displayed parameters have been synthesised in given ranges. Every blue region depicts a set of values satisfying the stated property in at least one initial value of model variables. In this particular case, the blue regions make joint projections across all dimensions (i.e., parameters and variables) of the modelled system. Consequently, the property is confirmed to be satisfied by every combination of parameters (respectively, initial conditions) in the considered ranges.

**Property** **4.** *Eventually, there will happen a situation where the population never exceeds a low value (stays below 0.08 g/L forever) and TCP concentration stays above 0.01 mM (never fully degrades).*

To support the above results, we decided to consider a property with the opposite meaning (Property 4). In particular, the analysis resulted with no positive results which shows us a certain agreement with the previous analysis. However, as we have already addressed, we cannot directly interpret an “empty result” due to potential existence of false-positives. Therefore, we decided to investigate Property 4 more deeply with the help of the robustness monitoring analysis (see the analysis of Property 7).

**Property** **5.** *Eventually, there will happen a situation in which TCP concentration is currently above 0.01 mM and the population never exceeds a low value (stays below 0.08 g/L forever).*

**Figure 13:**
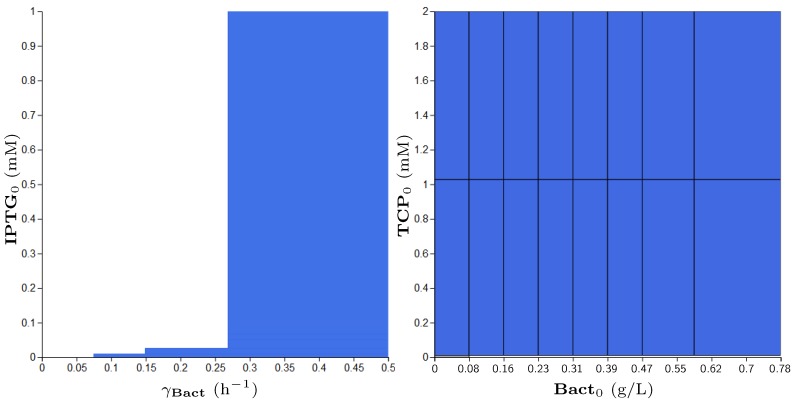
Results of parameter synthesis process for Property 5. Inside the figure, one can see two plots with blue regions. Both plots show a combination of parameters and (or) variables of the model where each point represents the particular evaluation of considered parameters (or variables). Every blue region represents a set of evaluations satisfying the stated property in at least one initial condition of the model. Here, the blue regions make joint projections across all non-displayed dimensions (i.e., parameters and variables). Consequently, the property holds in every combination of initial conditions (respectively, parameters) in the particular ranges.

An interesting fact appears in the analysis based on comparing Property 4 with its weaker form represented by Property 5. A difference between the two properties is only in the predicate about the TCP concentration. However, for the parameter synthesis method, the difference is significant; as in Property 5, we do not require the concentration of TCP to be above 0.01 mM forever. In Figure 13, it is shown that for the weaker property there exist positive results. However, they appear only for the population death rate (parameter $\gamma_{\mathrm{Bact}}$) higher than 0.07 $s^{-1}$, which is an overrated value.

The parameter synthesis method works with abstractions of systems dynamics that do not consider time explicitly. However, it allows exploring patterns of model dynamics globally (regardless of the concrete settings of initial conditions). On the contrary, the robustness monitoring method considers time but works locally (i.e., dynamics is simulated in time for a given set of initial conditions). Therefore, there is a chance of missing an interesting behaviour occurring for an initial condition which is not included in the considered set.

**Property** **6.** *The population will never drop below half of its initial value in the 5 h scope and TCP will degrade (drop below 0.01 mM) in the 2.5 h scope at the same time.*

**Figure 14:**
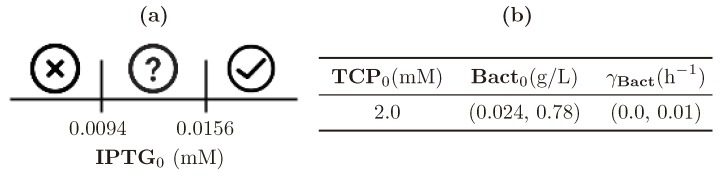
Diagram for satisfiability of Property 6 reflecting ${\mathrm{IPTG}}_{0}$. (**a**) A simple diagram presenting the qualitative results of robustness monitoring for Property 6. It shows the influence of ${\mathrm{IPTG}}_{0}$ on the satisfiability of the particular property. The two thresholds divide the area of influence into three sections. The left one which robustly violates the property, the right one—satisfying the property (robustly)—and the middle one where the result is not robust. This result holds for all combinations of the parameters (and variables) in the table (**b**).

**Figure 15:**
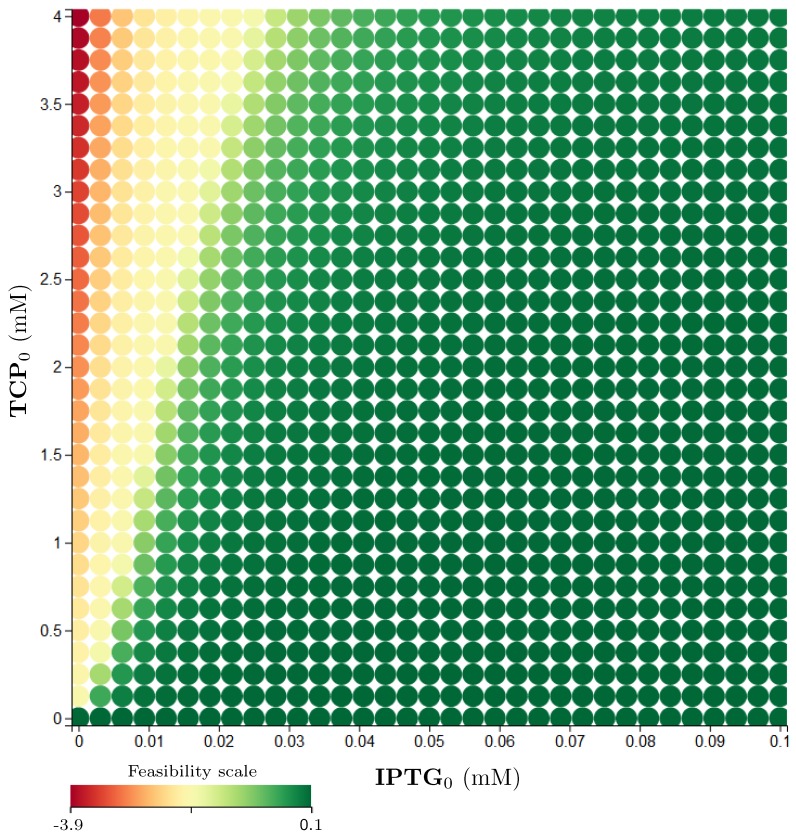
Results of robustness monitoring for Property 6 concerning ${\mathrm{TCP}}_{0}$. The figure shows a two-dimensional plot with various coloured circles pointing by their centre to the particular setting of the plotted parameters (or variables). Initial values of variables and considered parameters (if not displayed in any axis) are: ${\mathrm{Bact}}_{0}=0.487$ (g/L); ${\mathrm{GLY}}_{0}$, $(R{)-\mathrm{DCP}}_{0}$, $(S{)-\mathrm{DCP}}_{0}$, ${\mathrm{ECH}}_{0}$, ${\mathrm{CPD}}_{0}$, ${\mathrm{GDL}}_{0}$, ${\mathrm{TCP}}_{0}=0$ (mM); $\gamma_{\mathrm{Bact}}=0.0022$ $\left(h^{-1}\right)$. All the constants can be found in Figure 11. The shades of green colour imply a feasibility of the particular property in the particular initial setting while the shades of red imply a violation of the property—the darker the tone, the stronger the feasibility/violation. At the bottom of the plot, there is the feasibility scale mapped to real values. The plot represents a single layer of the entire parameter space.

Property 6 gives another reformulation of the desired property that now includes timing aspects. It requires the entire TCP degradation to be realised in a certain time limit. The particular results obtained with robustness monitoring are shown in Figure S10. For brevity, we present a simple diagram (Figure 14) showing that the change of the population death rate ($\gamma_{\mathrm{Bact}}$) as well as the initial size of the population (${\mathrm{Bact}}_{0}$) have practically no influence on this property. In Figure S11, the range of the death rate coefficient is increased to the number of 0.1 ($h^{-1}$), which is a considerably overrated value. However, the influence is still considered negligible. More interesting appears to be the effect of the TCP initial concentration (${\mathrm{TCP}}_{0}$) on the satisfiability of Property 6. The dependency between ${\mathrm{TCP}}_{0}$ and ${\mathrm{IPTG}}_{0}$ is non-linear (Figure 15).

**Property** **7.** *The population dies eventually (drops below 0.01 g/L) while TCP does not degrade entirely (does not drop below 0.1 mM) in the 5 h horizon.*

To justify the results of parameter synthesis obtained for Property 4, we formulate a similar property that is compatible with the robustness monitoring method (Property 7). The results are shown in Figure S12—they contain only negative values (the property is definitely not satisfied in any sampled point). The summary of these results is available in a compact table (Table 1). For the reasonable range of the death rate coefficient, the results do not change significantly. However, there is some noticeable influence for the increased range of the death rate (up to 0.1 $h^{-1}$) shown in Figure S13. Nevertheless, the obtained negative results support the previous outcome of parameter synthesis and even improve it by adding the quantitative information.

Robustness monitoring proves to be useful for this type of models. On the other hand, parameter synthesis approach is designed for models with a more complex relationship between variables where some interesting bifurcations can take place.

## 4. Conclusions

The effects of metabolites on the fitness of bacterial strains carrying artificial pathways are of great interest for the community of metabolic engineers. The adverse effects of toxic metabolites and metabolic burden on the host cells need to be considered and ideally even quantitatively characterised by the computational modelling. Here, we present development of such a model for the *E. coli* BL21(DE3) strain overexpressing the artificial pathway for degradation of highly toxic environmental pollutant 1,2,3-trichloropropane (TCP).

To assess the complex behaviour of the system, we created a unique mathematical model which combines population modeling with prediction of effects of metabolite toxicity and burden caused by application of the standard synthetic inducer IPTG triggering expression of heterologous biodegradation pathway. We were interested in conditions of the system in which the biodegradation is efficient while population survives and we investigated the system under these conditions using state-of-the-art computational methods.

First, we extended the original model of the metabolic pathway with a new model variable describing the bacterial population growth over time. The new model reflects the initial concentration of the inducer (${\mathrm{IPTG}}_{0}$). Second, we formalised the following features of the investigated system: (1) the metabolic burden effect caused by increased concentration of the inducer; (2) a highly toxic effect of TCP and other metabolites of the pathway; and (3) an exciting phenomenon of toxicity exacerbation occurred by the joint effect of the inducer (IPTG) and the pathway’s substrate (TCP). Finally, using computational biology methods, we investigated the continuous parameter space of initial conditions and uncertain coefficients targeting the interesting properties of the model. We believe that the obtained results give a solid basis for further optimisation of the synthetic pathway in the considered strain.

Further refinement of the model is planned for future work. In particular, we aim at producing the data describing increasing enzyme concentrations in time. That will allow us to generalise the model by representing enzyme concentrations as model variables. Although the model itself is fine-tuned with respect to the considered *E. coli* BL21(DE3) strain, it can provide a modelling basis in different scenarios where a non-metabolisable inducer is used to control a closed cell population environment in stationary phase. On the computational side, the employed computational framework combining the carefully selected set of formal methods and simulation-based tools can be reused in any modelling case fitting the class of non-linear ODEs including the enzyme kinetics.

## Figures and Tables

**Table 1:** Results of robustness monitoring for Property 7. A simple table presents the relevant ranges of initial conditions (i.e., the concentration of variables and setting of parameters) which robustly violate Property 7.

${\mathrm{IPTG}}_{0}\;\left(\mathrm{mM}\right)$	${\mathrm{TCP}}_{0}\left(\mathrm{mM}\right)$	${\mathrm{Bact}}_{0}\left(g/L\right)$	$\gamma_{\mathrm{Bact}}\;(h^{-1})$
(0.0, 1.0)	(0.0, 4.0)	(0.024, 0.78)	(0.0, 0.01)

**Figure A1:**
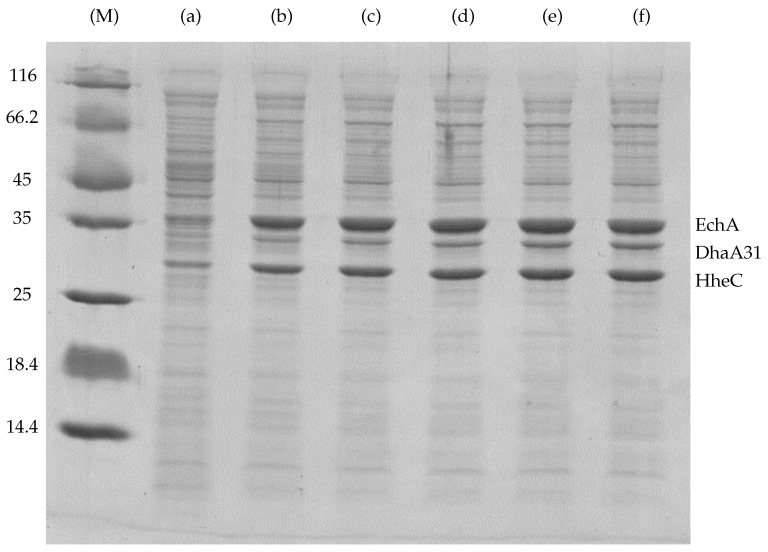
Sodium dodecyl sulfate polyacrylamide gel electrophoresis of cell-free extracts obtained from *E. coli* deg31 cells induced with various concentrations of IPTG ((**M**) protein marker (116, 66.2, 45, 35, 25, 18.4, and 14.4 kDa)): (**a**) 0.0 mM IPTG; (**b**) 0.01 mM IPTG; (**c**) 0.025 mM IPTG; (**d**) 0.05 mM IPTG; (**e**) 0.2 mM IPTG; and (**f**) 1.0 mM IPTG. Bands of DhaA31 (34 kDa), HheC (29 kDa) and EchA (35 kDa) are marked.

## Supplementary Materials

The following are available online at https://www.mdpi.com/2076-2607/7/11/553/s1, Data S1: IPTG growth curves; Data S2: Growth on 10 mM glycerol; Data S3: Toxicity on 10 mM glycerol; Figure S1: The comparative test of two mathematical models for biodegradation of TCP; Figure S2: The model of the metabolic pathway for biodegradation of TCP without reverse reactions; Figure S3: The comparative simulation of two bacterial growth functions; Figure S4: Evidence of need for proper function describing population growth reflecting metabolic burden caused by IPTG; Figure S5: The results of fitting to population growth data reflecting toxicity caused by TCP; Figure S6: The results of fitting to population growth data reflecting toxicity caused by ECH; Figure S7: The results of fitting to population growth data reflecting toxicity caused by CPD; Figure S8: The results of fitting to population growth data reflecting toxicity caused by GDL; Figure S9: The results of fitting to population growth data reflecting toxicity caused by TCP—prolonged simulation; Figure S10: The results of robustness monitoring for Property 6; Figure S11: The results of robustness monitoring for Property 6 with extended range of the death rate coefficient; Figure S12: The results of robustness monitoring for Property 7; Figure S13: The results of robustness monitoring for Property 7 with extended range of the death rate coefficient; Table S1: The quantitative results for fitting of the functions explaining the enzymes concentration reflecting IPTG; and Table S2: The results of fitting several growth functions to the same set of experimental data.

## Abbreviations

The following abbreviations are used in this manuscript:

IPTG	isopropyl-beta-D-thiogalactopyranoside
TCP	1,2,3-trichloropropane
DCP	2,3-dichloropropane-1-ol
ECH	epichlorohydrin
CPD	3-chloropropane-1,2-diol
GDL	glycidol
GLY	glycerol
DhaA	haloalkane dehalogenase
EchA	epoxide hydrolase
HheC	haloalcohol dehalogenase
MM	Michaelis-Menten
CDW	cell dry weight
ODE	ordinary differential equations
MCMC	Markov chain Monte Carlo
GUI	graphical user interface
CLI	command-line interface

## Appendix A. The Experimental Data Used for the Fitting of Functions Explaining the Enzymes Concentration Determined at Respective IPTG Concentrations

In Table A1 and Table A2, the results of the experiments published in [19] are presented. These results contain data from two experiments, one of which is shown in Figure A1. The tables show relative portions of enzymes in total soluble protein content of cell-free extract for different starting concentrations of IPTG—the inducer used for induction of host cells (*E. coli* deg31) under conditions described in Laboratory Methods.

The total masses of enzymes $\left(\frac{\mathrm{mg}}{10\;\mathrm{mL}}\right)$ were calculated from value 4.02 (measured for 0.2 mM IPTG and coloured in blue) appropriately for the rest of IPTG concentrations reflecting different portions of total enzymes (Column 2 in Table A1 and Table A2). The total mass values were then used for calculation of the enzymes mass in cell-free extract (Table A3 and Table A4) with respect to the particular enzyme relative portion (Table A1 and Table A2, respectively). Then, using atomic mass values in Table A5 and enzyme masses in cell-free extract from two different experiments, we calculated molar concentrations (mM) for all enzymes. Finally, the median and standard deviation were calculated from these values (Table A6) and used for fitting, as described in Section 3.3.

**Table A1:** Relative fractions of enzymes from Experiment 1.

IPTG (mM)	Content in Cell-Free Extract (%) *	DhaA31 (Relative Portion)	HheC (Relative Portion)	EchA (Relative Portion)	Total Mass ($\frac{\mathrm{mg}}{10\;\mathrm{mL}}$)
1.0	50	0.19	0.39	0.42	4.188
0.2	48	0.17	0.40	0.43	4.02
0.05	40	0.13	0.38	0.49	3.350
0.025	40	0.12	0.41	0.47	3.350
0.01	22	0.16	0.36	0.48	1.843
0.0	15	0.20	0.33	0.47	1.256

* Calculated as a portion of total soluble protein content.

**Table A2:** Relative fractions of enzymes from Experiment 2.

IPTG (mM)	Content in Cell-Free Extract (%) *	DhaA31 (Relative Portion)	HheC (Relative Portion)	EchA (Relative Portion)	Total Mass ($\frac{\mathrm{mg}}{10\;\mathrm{mL}}$)
1.0	60	0.15	0.39	0.46	3.890
0.2	62	0.15	0.39	0.46	4.020
0.05	50	0.13	0.38	0.49	3.242
0.025	50	0.11	0.37	0.52	3.242
0.01	42	0.11	0.37	0.52	2.723
0.0	15	0.17	0.40	0.44	0.973

* Calculated as a portion of total soluble protein content.

**Table A3:** Mass and molar concentration of enzymes in cell-free extract from Experiment 1.

IPTG	Total Mass	DhaA31	HheC	EchA	DhaA31	HheC	EchA
(mM)	$\left(\frac{\mathrm{mg}}{10\;\mathrm{mL}}\right)$	$\left(\frac{\mathrm{mg}}{L}\right)$ *	$\left(\frac{\mathrm{mg}}{L}\right)$ *	$\left(\frac{\mathrm{mg}}{L}\right)$ *	(mM) ^#^	(mM) ^#^	(mM) ^#^
1.0	4.188	79.6	163.3	175.9	2.24 × 10^−3^	5.57 × 10^−3^	4.82 × 10^−3^
0.2	4.02	68.3	160.8	172.9	1.92 × 10^−3^	5.48 × 10^−3^	4.74 × 10^−3^
0.05	3.350	43.6	127.3	164.2	1.22 × 10^−3^	4.34 × 10^−3^	4.50 × 10^−3^
0.025	3.350	40.2	137.4	157.5	1.13 × 10^−3^	4.68 × 10^−3^	4.32 × 10^−3^
0.01	1.843	29.5	66.3	88.4	8.29 × 10^−4^	2.26 × 10^−3^	2.43 × 10^−3^
0.0	1.256	25.1	41.5	59.0	7.06 × 10^−4^	1.41 × 10^−3^	1.62 × 10^−3^

* Calculated as a relative portion of the total mass $\left(\frac{\mathrm{mg}}{10\;\mathrm{mL}}\right)\times 100$. ^#^ Calculated as $\left(\frac{\mathrm{mg}}{\mathrm{Da}\;\cdot \;L}\right)$.

**Table A4:** Mass and molar concentration of enzymes in cell-free extract from Experiment 2.

IPTG	Total Mass	DhaA31	HheC	EchA	DhaA31	HheC	EchA
(mM)	$\left(\frac{\mathrm{mg}}{10\;\mathrm{mL}}\right)$	$\left(\frac{\mathrm{mg}}{L}\right)$ *	$\left(\frac{\mathrm{mg}}{L}\right)$ *	$\left(\frac{\mathrm{mg}}{L}\right)$ *	(mM) ^#^	(mM) ^#^	(mM) ^#^
1.0	3.89	58.4	151.7	179.0	1.64 × 10^−3^	5.17 × 10^−3^	4.91 × 10^−3^
0.2	4.02	60.3	156.8	184.9	1.69 × 10^−3^	5.34 × 10^−3^	5.07 × 10^−3^
0.05	3.242	42.1	123.2	158.9	1.18 × 10^−3^	4.20 × 10^−3^	4.36 × 10^−3^
0.025	3.242	35.7	120.0	168.6	1.00 × 10^−3^	4.09 × 10^−3^	4.62 × 10^−3^
0.01	2.723	30.0	100.8	141.6	8.42 × 10^−4^	3.44 × 10^−3^	3.88 × 10^−3^
0.0	0.973 × 10^−1^	16.5	38.9	42.8	4.65 × 10^−4^	1.33 × 10^−3^	1.17 × 10^−3^

* Calculated as a relative portion of the total mass $\left(\frac{\mathrm{mg}}{10\;\mathrm{mL}}\right)\times 100$. ^#^ Calculated as $\left(\frac{\mathrm{mg}}{\mathrm{Da}\;\cdot \;L}\right)$.

**Table A5:** Mass values of enzymes.

DhaA31 (Da)	HheC (Da)	EchA (Da)
35,576.37	29,333.07	36,465.11

**Table A6:** Molar concentration of enzymes in cell-free extract calculated as a median of the values in Table A3 and Table A4 with standard deviation values (i.e., stdev columns).

IPTG	DhaA31 (mM)		HheC (mM)		EchA (mM)	
(mM)	Median	stdev	Median	stdev	Median	stdev
1.0	1.94 × 10^−3^	4.22 × 10^−4^	5.37 × 10^−3^	2.79 × 10^−4^	4.87 × 10^−3^	5.97 × 10^−5^
0.2	1.81 × 10^−3^	1.60 × 10^−4^	5.41 × 10^−3^	9.69 × 10^−5^	4.91 × 10^−3^	2.34 × 10^−4^
0.05	1.20 × 10^−3^	2.78 × 10^−5^	4.27 × 10^−3^	9.90 × 10^−5^	4.43 × 10^−3^	1.03 × 10^−4^
0.025	1.07 × 10^−3^	9.02 × 10^−5^	4.39 × 10^−3^	4.19 × 10^−4^	4.47 × 10^−3^	2.16 × 10^−4^
0.01	8.35 × 10^−4^	9.45 × 10^−6^	2.85 × 10^−3^	8.30 × 10^−4^	3.15 × 10^−3^	1.03 × 10^−3^
0.0	5.85 × 10^−4^	1.71 × 10^−4^	1.37 × 10^−3^	6.15 × 10^−5^	1.40 × 10^−3^	3.15 × 10^−4^

## Appendix B. The Origin of the Death Rate Coefficient

We used the mean value of death rate in percentage per generation (% p.g.) from article *Robust growth of Escherichia coli* [68] for *E. coli* strain B/r, which is close to the BL21 we used in experiments. The particular value ranges from 0.5% p.g. to 1% p.g.. However, we need a death rate per hour ($h^{-1}$) in our model. To achieve that, we divided p.g. values with the generation (doubling) time of the population in our model. The growth rate (g) in our model is in the range [0.07,0.26] ($h^{-1}$) according to fitting of the model to the experimental data. Thus, the particular values of generation time ($t_{g}$) are between 2.58 h and 9.29 h for the doubling of the population. Both growth rate and doubling time were obtained as a median from at least three experiments. The final death-rate-per-hour values—limiting the range—are the maximum and minimum (emphasised numbers) from Table A7.

**Table A7:** The death rate (γ) coefficients evaluated from different growth rates (g) using the generation time ($t_{g}$) and percentage death rate per generation (% p.g.).

g ($h^{-1}$)	$t_{g}$ (h)	γ ($h^{-1}$) as 0.5% p.g.	γ ($h^{-1}$) as 1% p.g.
0.26	2.58	$1.938\;\times \;10^{-3}$	$3.876\;\times \;10^{-3}$
0.07	9.29	$5.382\;\times \;10^{-4}$	$1.076\;\times \;10^{-3}$

## Appendix C. The List of Considered Inhibition Models

In this step of modelling, we found a good model describing the bacterial population growth on a substrate (e.g., glycerol). In this case study, the model involves traditional Monod equation [28] as the growth rate function:

(A1) $$\mu =\frac{\mu_{\max}\left[S\right]}{K_{S}+\left[S\right]}$$

where μ is the specific growth rate, $\mu_{\max}$ is the maximum specific growth rate for the culture, [S] is the substrate concentration, and $K_{S}$ is the *half-saturation constant* (or the *affinity constant*). Both $\mu_{\max}$ and $K_{S}$ reflect intrinsic physiological properties of a particular type of microorganism, the substrate utilisation and the temperature of growth ([67], Chapter 3).

The Monod equation can express the rate of change in the number of cells as well as the fluxion of the cell mass (both can be defined by [B]). Thus, the whole model of bacterial growth is:

(A2) $$\frac{d[B]}{dt}=\left[B\right]\;\cdot \;\frac{\mu_{\max}\left[S\right]}{K_{S}+\left[S\right]}$$

In general, we could use a different model as the growth rate function [52]. We only need to have some proper model and think of it as an abstract module describing population growth. In this way, we can use a different module to explain the inhibition of the population growth by a poisonous substance (e.g., a toxic water pollutant such as 1,2,3-trichloropropane, TCP). However, there is no universal inhibition model. Therefore, we conducted several simulations with various combinations of models to fit the experimental data properly. In the following list, [B], [S] and [X] stand for the bacterial population, the substrate concentration and the toxic substance concentration, respectively. The majority of the following equations consist of a growth module (e.g., Monod) and some inhibition module (separated by minus character). Equations (11)–(16) adopt a different form (in general called *competitive inhibition* [25]) using one module (e.g., Monod) extended by different types of inhibition:

Monod + Hill [27]:	$\frac{d[B]}{dt}=\left[B\right]\;\cdot \;\frac{\mu_{\max}\left[S\right]}{K_{S}+\left[S\right]}-\left[B\right]\;\cdot \;\frac{k{\left[X\right]}^{n}}{{K_{i}}^{n}+{\left[X\right]}^{n}}$	(A3)
Monod + Aiba-Edward [65]:	$\frac{d[B]}{dt}=\left[B\right]\;\cdot \;\frac{\mu_{\max}\left[S\right]}{K_{S}+\left[S\right]}-\left[B\right]\;\cdot \;\frac{k[X]}{K_{X}+\left[X\right]}\;\cdot \;\exp\left(\frac{-[X]}{K_{i}}\right)$	(A4)
Monod + Andrews [66]:	$\frac{d[B]}{dt}=\left[B\right]\;\cdot \;\frac{\mu_{\max}\left[S\right]}{K_{S}+\left[S\right]}-\left[B\right]\;\cdot \;\frac{k}{\left(1+\frac{K_{X}}{\left[X\right]}\right)\left(1+\frac{\left[X\right]}{K_{i}}\right)}$	(A5)
Monod + Haldane-Andrews [66]:	$\frac{d[B]}{dt}=\left[B\right]\;\cdot \;\frac{\mu_{\max}\left[S\right]}{K_{S}+\left[S\right]}-\left[B\right]\;\cdot \;\frac{k[X]}{K_{X}+\left[X\right]+\frac{{\left[X\right]}^{2}}{K_{i}}}$	(A6)
Monod + Monod:	$\frac{d[B]}{dt}=\left[B\right]\;\cdot \;\frac{\mu_{\max}\left[S\right]}{K_{S}+\left[S\right]}-\left[B\right]\;\cdot \;\frac{k[X]}{K_{X}+\left[X\right]}$	(A7)
Monod + Moser [64]:	$\frac{d[B]}{dt}=\left[B\right]\;\cdot \;\frac{\mu_{\max}\left[S\right]}{K_{S}+\left[S\right]}-\left[B\right]\;\cdot \;\frac{k{\left[X\right]}^{n}}{K_{X}+{\left[X\right]}^{n}}$	(A8)
Monod + Tessier [63]:	$\frac{d[B]}{dt}=\left[B\right]\;\cdot \;\frac{\mu_{\max}\left[S\right]}{K_{S}+\left[S\right]}-\left[B\right]\;\cdot \;k\left(1-\exp\left(\frac{-[X]}{K_{i}}\right)\right)$	(A9)
Monod + Tessier-type [65]:	$\frac{d[B]}{dt}=\left[B\right]\;\cdot \;\frac{\mu_{\max}\left[S\right]}{K_{S}+\left[S\right]}-\left[B\right]\;\cdot \;k\left(\exp\left(\frac{-[X]}{K_{i}}\right)-\exp\left(\frac{-[X]}{K_{X}}\right)\right)$	(A10)
competitive inhibition:	$\frac{d[B]}{dt}=\left[B\right]\;\cdot \;\frac{\mu_{\max}\left[S\right]}{K_{S}\left(1+\frac{\left[X\right]}{K_{i}}\right)+\left[S\right]}$	(A11)
non-competitive inhibition:	$\frac{d[B]}{dt}=\left[B\right]\;\cdot \;\frac{\mu_{\max}}{1+\frac{\left[X\right]}{K_{i}}}\;\cdot \;\frac{\left[S\right]}{K_{S}+\left[S\right]}$	(A12)
uncompetitive inhibition:	$\frac{d[B]}{dt}=\left[B\right]\;\cdot \;\frac{\mu_{\max}\left[S\right]}{K_{S}+\left[S\right]\left(1+\frac{\left[X\right]}{K_{i}}\right)}$	(A13)
non-competitive inhibition using negative Hill	$\frac{d[B]}{dt}=\left[B\right]\;\cdot \;\frac{\mu_{\max}\left[S\right]}{K_{S}+\left[S\right]}\;\cdot \;\frac{k\;\cdot \;{K_{i}}^{n}}{{K_{i}}^{n}+{\left[X\right]}^{n}}$	(A14)
non-competitive inhibition using negative Moser	$\frac{d[B]}{dt}=\left[B\right]\;\cdot \;\frac{\mu_{\max}\left[S\right]}{K_{S}+\left[S\right]}\;\cdot \;\frac{k\;\cdot \;K_{i}}{K_{i}+{\left[X\right]}^{n}}$	(A15)
non-competitive exponential inhibition	$\frac{d[B]}{dt}=\left[B\right]\;\cdot \;\frac{\mu_{\max}\left[S\right]}{K_{S}+\left[S\right]}\;\cdot \;\frac{k}{{K_{i}}^{\left[X\right]}}$	(A16)

where $K_{i}$ and $K_{X}$ are inhibition constants explaining inhibitory effects of poisonous substance at higher concentrations; *k* is an inhibitory rate; and *n* is a coefficient explaining cooperativity (as defined in Hill equation) or another biochemical property, depending on the context.
