# Supplementary material for: Computational Modelling of Metabolic Burden and Substrate Toxicity in Escherichia coli Carrying a Synthetic Metabolic Pathway
Source: Microorganisms. 2019 Nov 11;7(11):553. doi: 10.3390/microorganisms7110553 (PMC6921056; doi:10.3390/microorganisms7110553)
Supplement: Supplementary file 1 [file microorganisms-07-00553-s001.zip › microorganisms-618432-SI.pdf]

Table S1: **The quantitative results for fitting of the functions explaining the enzymes concentration reflecting IPTG.**
The particular results (Best-fit) along with statistical evaluation (R squared, Sum of squares, 95% confidence interval) are summarised in the table.

| Enzyme | Parameter | Best-fit | $R^2$ | Sum of squares | 95% CI |
|---|---|---|---|---|---|
| DhaA | $V_{\text{max,D}}$ | $1.9 \cdot 10^{-3}$ | 0.731 | $3.83 \cdot 10^{-7}$ | $[1.24, 2.73] \cdot 10^{-3}$ |
| | $K_{\text{M,D}}$ | $1.75 \cdot 10^{-2}$ | | | $[2.48, 805.4] \cdot 10^{-4}$ |
| HheC | $V_{\text{max,H}}$ | $5.39 \cdot 10^{-3}$ | 0.819 | $2.12 \cdot 10^{-6}$ | $[4.01, 6.98] \cdot 10^{-3}$ |
| | $K_{\text{M,H}}$ | $8.26 \cdot 10^{-3}$ | | | $[-\infty, 2.91 \cdot 10^{-2}]$ |
| EchA | $V_{\text{max,E}}$ | $5 \cdot 10^{-3}$ | 0.793 | $2 \cdot 10^{-6}$ | $[3.75, 6.41] \cdot 10^{-3}$ |
| | $K_{\text{M,E}}$ | $4.86 \cdot 10^{-3}$ | | | $[-\infty, 1.97 \cdot 10^{-2}]$ |

Table S2: **The results of fitting several growth functions to the same set of experimental data.** The columns show the following: names of functions, number of parameters to fit (PTF), information whether fitting was successful, the multicollinearity factor for all parameters of the particular function meaning the linear dependency (or correlation) between parameters (higher values tend fitting to be inaccurate or even impossible), the sum of squared residuals (SSR) meaning the accumulation of discrepancy betweeen the experimental data and the fitted model (smaller values mean a better result).

| Function | PTF | Finished | Collinearity | SSR |
|---|---|---|---|---|
| Aiba–Edward | 4 | no | 252737.6 | unknown |
| Andrews | 4 | no | 848093 | unknown |
| Haldane–Andrews | 4 | no | 63500.8 | unknown |
| **Monod** | 3 | **yes** | 2.76 | 1237.536 |
| Moser | 4 | no | 126.5 | unknown |
| **Tessier** | 3 | **yes** | 2.84 | 1005.541 |
| Tessier II | 4 | no | unknown | unknown |

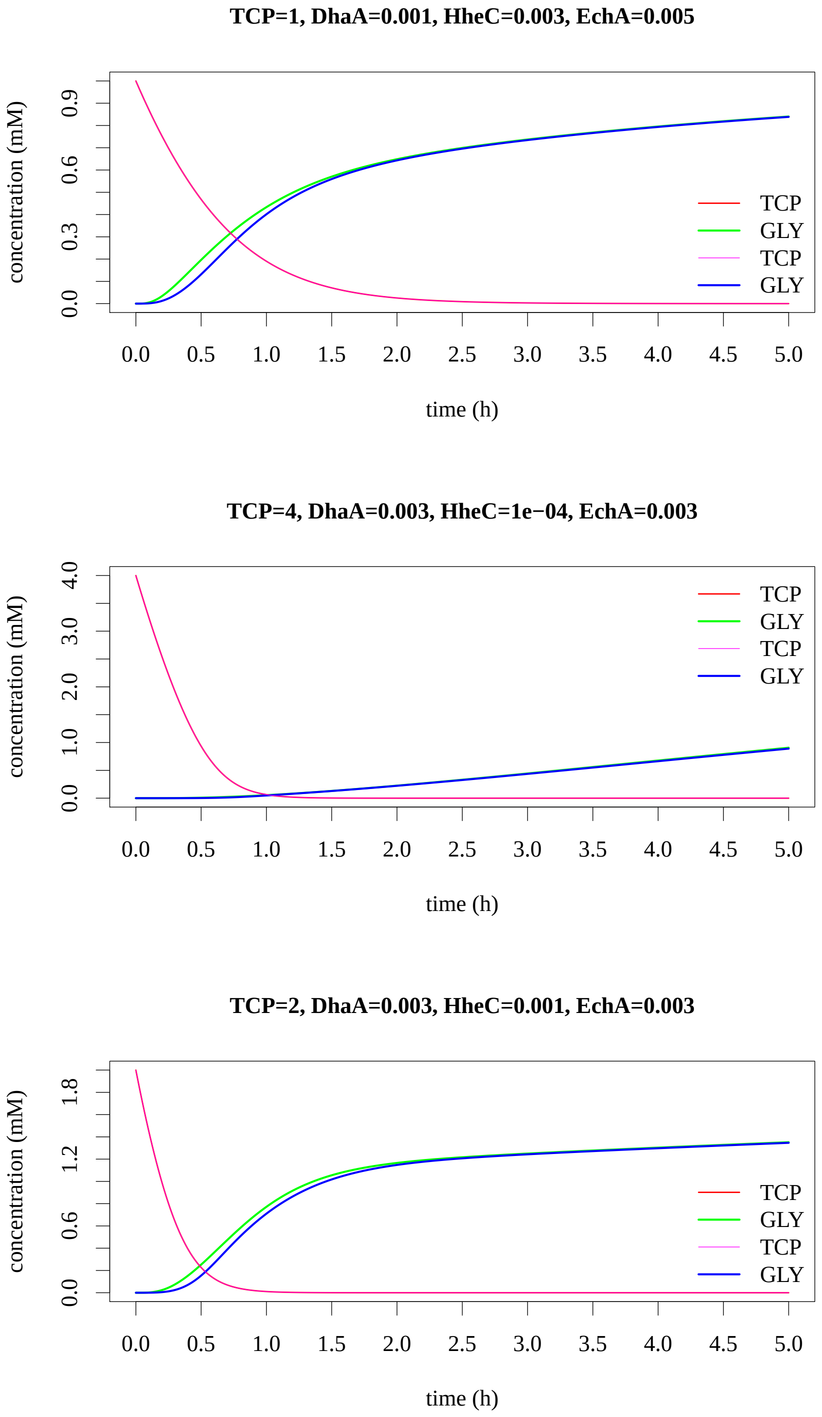


Figure S1: **The comparative test of two mathematical models for biodegradation of TCP.** Three figures comparing two slightly diferent mathematical models for the same problem. Note the relation between green and blue line describing the concentration of the product of the biodegradation pathway (GLY) in compared models. TCP (red and magenta) is the same in both models. The original model (with blue GLY line) contains competitive Michaelis-Menten function in one reaction while the other model (with green GLY line) uses ordinary Michaelis-Menten functions. Even under various conditions (i.e., input concentrations of the enzymes and TCP), the compared models show good-matching results.

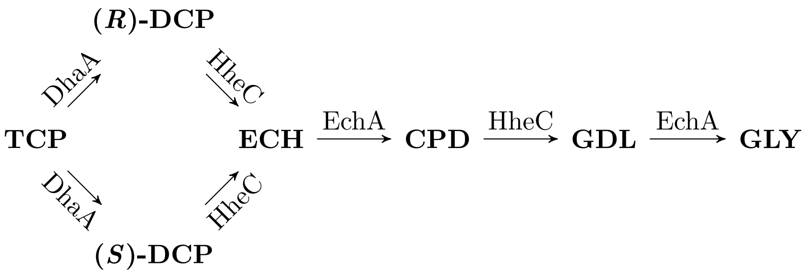


$$\frac{d[\mathrm{TCP}]}{dt} = -\frac{k_{1_R} \cdot \mathrm{DhaA} \cdot [\mathrm{TCP}]}{K_{m,1} + [\mathrm{TCP}]} - \frac{k_{1_S} \cdot \mathrm{DhaA} \cdot [\mathrm{TCP}]}{K_{m,1} + [\mathrm{TCP}]}$$

$$\frac{d[(R)\text{-DCP}]}{dt} = \frac{k_{1_R} \cdot \mathrm{DhaA} \cdot [\mathrm{TCP}]}{K_{m,1} + [\mathrm{TCP}]} - \frac{k_{2_R} \cdot \mathrm{HheC} \cdot [(R)\text{-DCP}]}{K_{m,2_R} + [(R)\text{-DCP}]}$$

$$\frac{d[(S)\text{-DCP}]}{dt} = \frac{k_{1_S} \cdot \mathrm{DhaA} \cdot [\mathrm{TCP}]}{K_{m,1} + [\mathrm{TCP}]} - \frac{k_{2_S} \cdot \mathrm{HheC} \cdot [(S)\text{-DCP}]}{K_{m,2_S} + [(S)\text{-DCP}]}$$

$$\frac{d[\mathrm{ECH}]}{dt} = \frac{k_{2_R} \cdot \mathrm{HheC} \cdot [(R)\text{-DCP}]}{K_{m,2_R} + [(R)\text{-DCP}]} + \frac{k_{2_S} \cdot \mathrm{HheC} \cdot [(S)\text{-DCP}]}{K_{m,2_S} + [(S)\text{-DCP}]} - \frac{k_3 \cdot \mathrm{EchA} \cdot [\mathrm{ECH}]}{K_{m,3} + [\mathrm{ECH}]}$$

$$\frac{d[\mathrm{CPD}]}{dt} = \frac{k_3 \cdot \mathrm{EchA} \cdot [\mathrm{ECH}]}{K_{m,3} + [\mathrm{ECH}]} - \frac{k_4 \cdot \mathrm{HheC} \cdot [\mathrm{CPD}]}{K_{m,4} + [\mathrm{CPD}]}$$

$$\frac{d[\mathrm{GDL}]}{dt} = \frac{k_4 \cdot \mathrm{HheC} \cdot [\mathrm{CPD}]}{K_{m,4} + [\mathrm{CPD}]} - \frac{k_5 \cdot \mathrm{EchA} \cdot [\mathrm{GDL}]}{K_{m,5} + [\mathrm{GDL}]}$$

$$\frac{d[\mathrm{GLY}]}{dt} = \frac{k_5 \cdot \mathrm{EchA} \cdot [\mathrm{GDL}]}{K_{m,5} + [\mathrm{GDL}]}$$

$$k_{1_R} = 0.58,\ k_{1_S} = 0.47,\ k_{2_R} = 1.81,\ k_{2_S} = 0.08,\ k_3 = 14.37,\ k_4 = 2.38,\ k_5 = 3.96,$$
$$K_{m,1} = 1.79,\ K_{m,2_R} = 2.49,\ K_{m,2_S} = 3.33,\ K_{m,3} = 0.09,\ K_{m,4} = 0.86,\ K_{m,5} = 3.54$$

Figure S2: **The model of the metabolic pathway for biodegradation of TCP without reverse reactions.**
A general scheme of the enzymatic chain reaction for biodegradation of TCP into GLY. The HheC enzyme is catalysing a reversible enzymatic reaction. However, in this particular case the *catalytic efficiency* (i.e., $\frac{k}{K_m}$) of EchA in turning ECH into CPD is much greater than the catalytic efficiency of HheC towards the reaction ECH → (R,S)-DCP. HheC also catalyses the reaction CPD ↔ GDL. The reverse reaction is compensated by a special kind of competitive version of Michaelis-Menten equation in the reaction GDL → GLY of the mathematical model. However, we conducted several comparative simulations as shown in Figure S1 which demonstrated futility of special MM function. Thus, the reverse reactions were removed. The mathematical model in forms of the ODE system is shown in the bottom half of the figure. Enzyme concentrations are considered as constants. Units: $k_x$ ($s^{-1}$) (as rate constants), $K_{m,x}$ (mM) (as Michaelis constants).

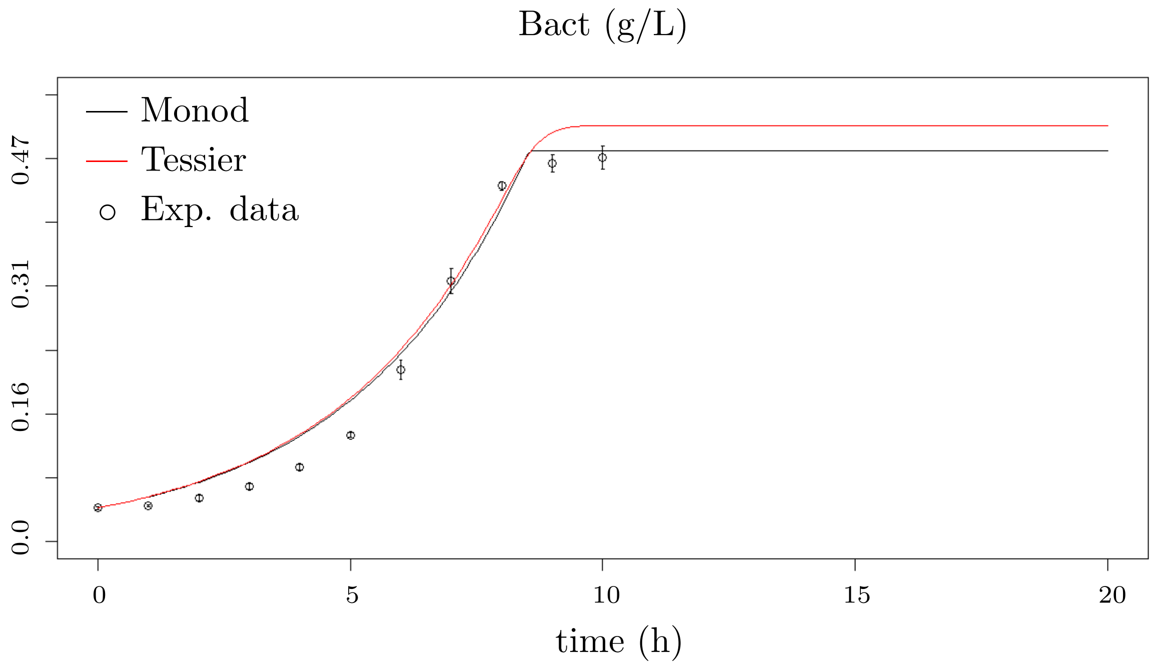


Figure S3: **The comparative simulation of two bacterial growth functions.**
Comparison of fitting results for Tessier model (red curve) and Monod model (black curve). Although, Tessier model shows better SSR (the sum of squared residuals — meaning the accumulation of discrepancy betweeen the experiments and the model) in Table S2, the result of simulation using Monod model is visually closer to the experimental data (circles) in the last time point.

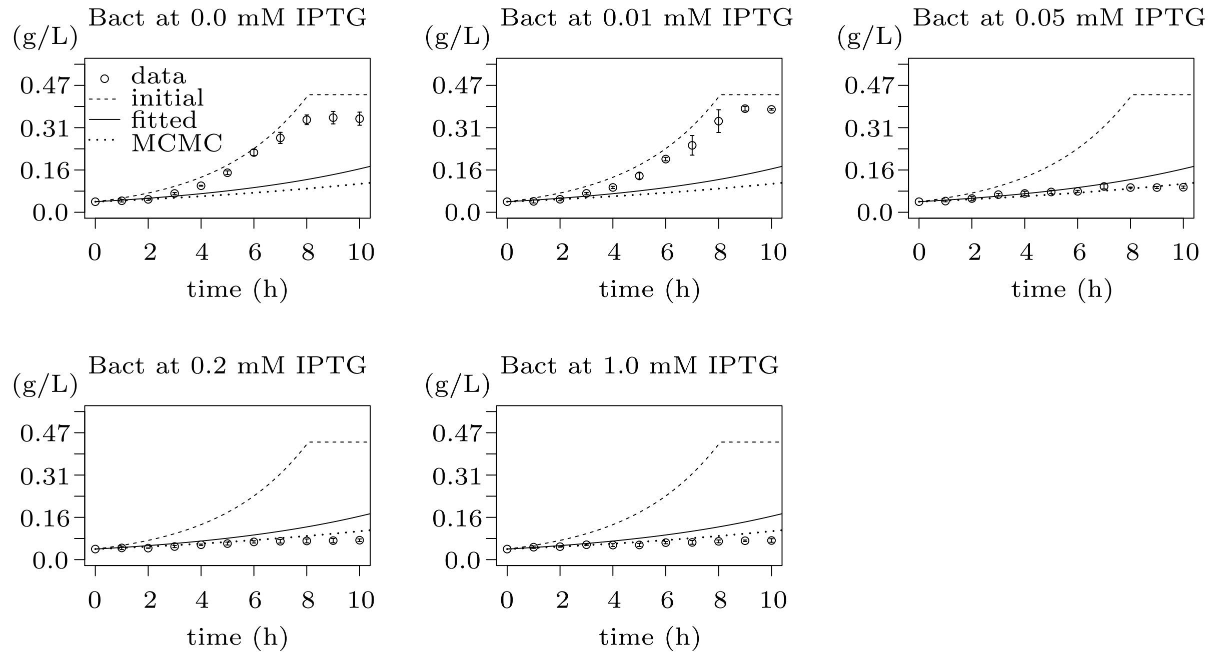


Figure S4: **Evidence of need for proper function describing population growth reflecting metabolic burden caused by IPTG.**

The plot contains five figures, each showing fitting results of the same model (Monod function) to the bacterial population growth data for different concentration of the inducer (IPTG) during 10 hours long induction phase reflecting the metabolic burden effect caused by increased concentration of IPTG. Apparently, used model does not reflect experimental data displayed as points with standard error bars. The dashed lines show simulation data for initial values of the model function (i.e., initial point of fitting). The solid lines show the results of non-linear regression and the dotted lines represent the final results optimised by Markov chain Monte Carlo (i.e., MCMC) method of the FME package. The x-axes show the time of experiment in hours while the y-axes show the bacterial population CDW in g/L.

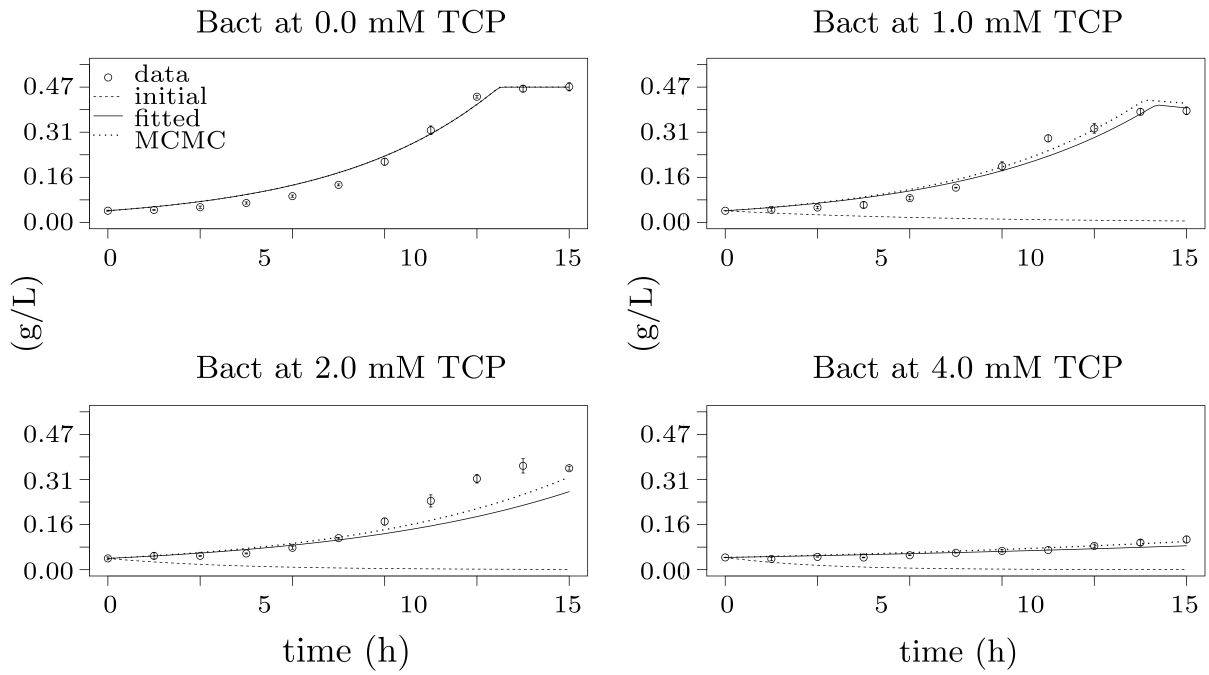


Figure S5: **The results of fitting to population growth data reflecting toxicity caused by TCP.** The plot contains four figures, each showing fitting of the same model to the bacterial population growth data for different concentration of the pathway substrate (TCP) for 10 hours. The experimental data are pictured as points with standard error bars, the dashed lines show simulation data for initial values of the model function (i.e., initial point of fitting), the solid lines show the results of non-linear regression (Material and Methods) and the dotted lines represent the final results optimised by MCMC method of the FME package which show the best agreement with the experimental data. The model with the best fit appears to be a combination of Monod function (describing the traditional bacterial growth) and Moser function (describing the degradation of the population caused by TCP presence). The x-axes show the time of experiment in hours while the y-axis of the plots show the bacterial population in g/L of cell dry weight.

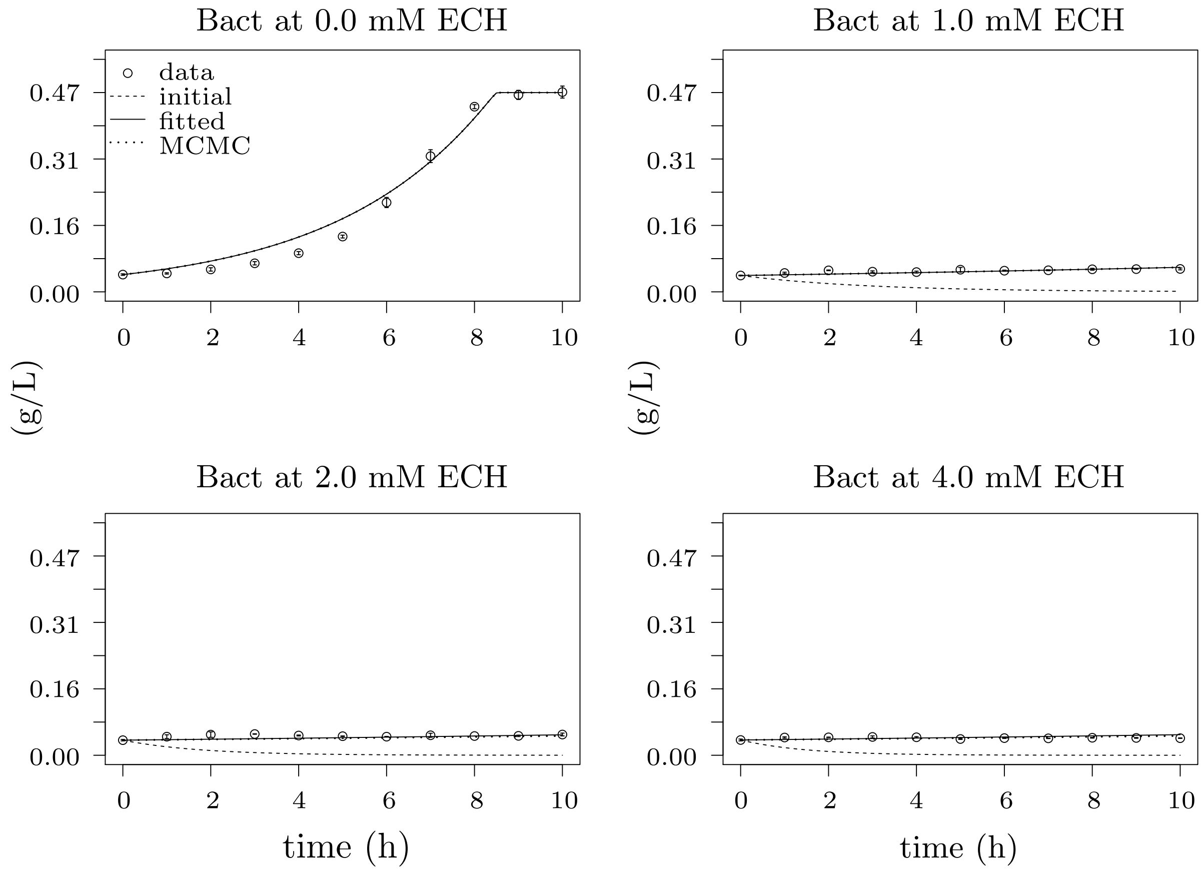


Figure S6: **The results of fitting to population growth data reflecting toxicity caused by ECH.** The plot contains four figures, each showing fitting of the same model to the bacterial population growth data for different concentration of the pathway intermediate product (ECH) for 10 hours. Apparently, the toxicity of ECH on the *E. coli* population is the most notable. The experimental data are pictured as points with standard error bars, the dashed lines show simulation data for initial values of the model function (i.e., initial point of fitting), the solid lines show the results of non-linear regression (Materials and Methods) and the dotted lines represent the final results optimised by MCMC method of the FME package which show the best agreement with the experimental data. The model with the best fit appears to be a combination of Monod function (describing the traditional bacterial growth) and Tessier function (describing the degradation of the population caused by ECH presence). The x-axes show the time of experiment in hours while the y-axis of the plots show the bacterial population in g/L of cell dry weight.

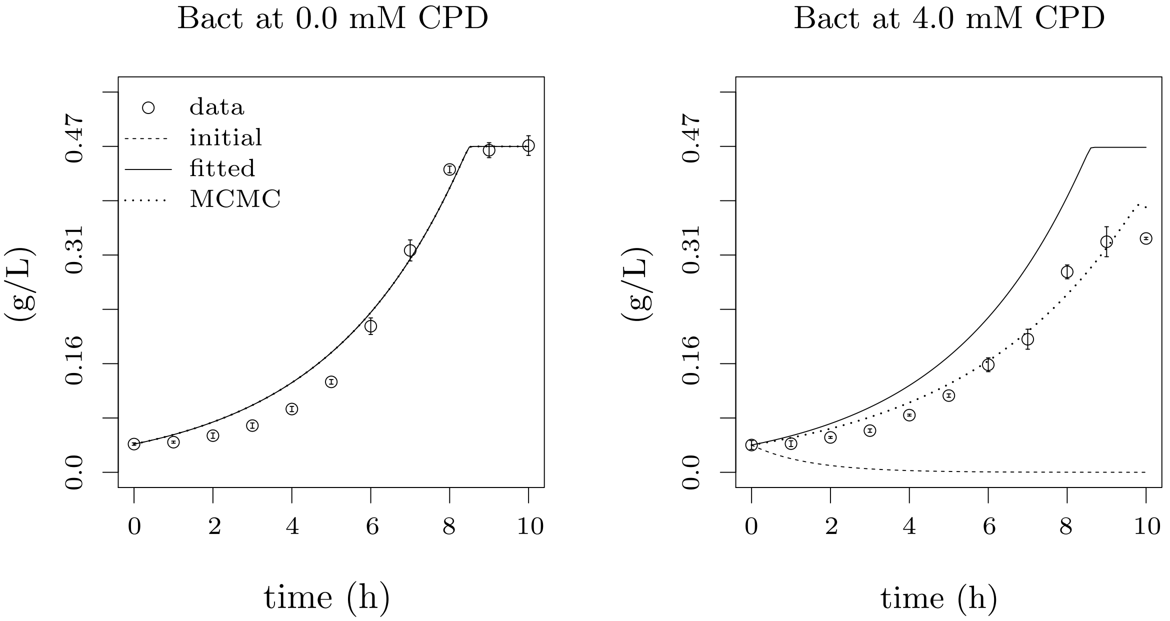


Figure S7: **The results of fitting to population growth data reflecting toxicity caused by CPD.** The plot contains two figures, each showing fitting of the same model to the bacterial population growth data for different concentration of the pathway intermediate product (CPD) for 10 hours. The experimental data are pictured as points with standard error bars, the dashed lines show simulation data for initial values of the model function (i.e., initial point of fitting), the solid lines show the results of non-linear regression (Materials and Methods) and the dotted lines represent the final results optimised by MCMC method of the FME package which show the best agreement with the experimental data. The model with the best fit appears to be a combination of Monod function (describing the traditional bacterial growth) and Tessier function (describing the degradation of the population caused by CPD presence). The x-axes show the time of experiment in hours while the y-axis of the plots show the bacterial population in g/L of cell dry weight.

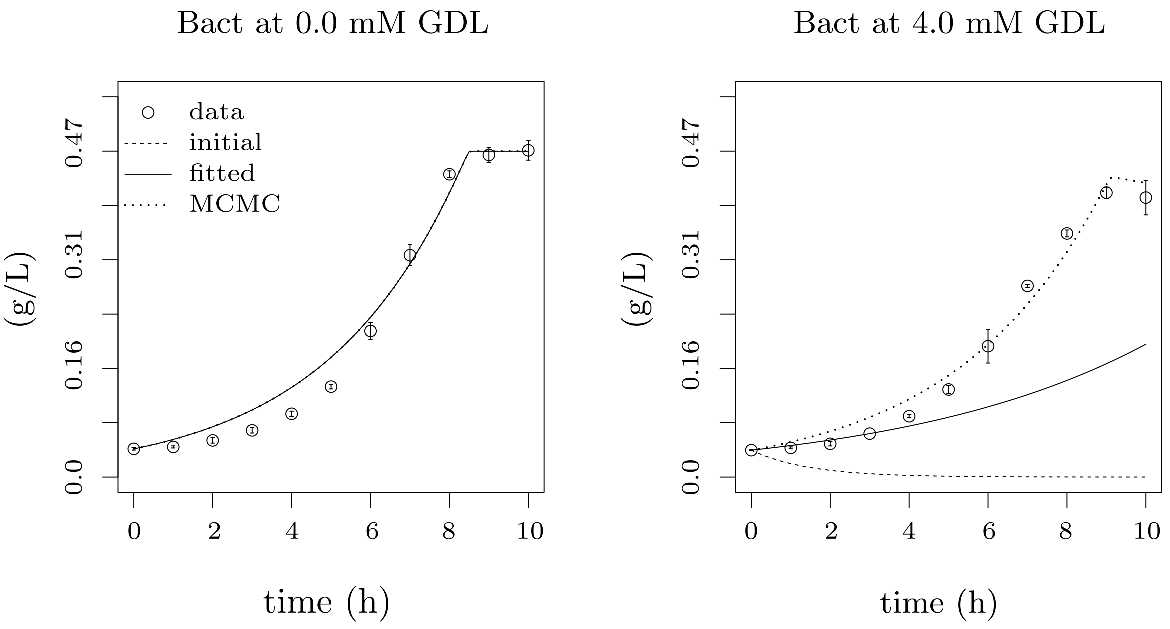


Figure S8: **The results of fitting to population growth data reflecting toxicity caused by GDL.** The plot contains two figures, each showing fitting of the same model to the bacterial population growth data for different concentration of the pathway intermediate product (GDL) for 10 hours. The experimental data are pictured as points with standard error bars, the dashed lines show simulation data for initial values of the model function (i.e., initial point of fitting), the solid lines show the results of non-linear regression (Materials and Methods) and the dotted lines represent the final results optimised by MCMC method of the FME package which show the best agreement with the experimental data. The model with the best fit appears to be a combination of Monod function (describing the traditional bacterial growth) and Tessier function (describing the degradation of the population caused by GDL presence). The x-axes show the time of experiment in hours while the y-axis of the plots show the bacterial population in g/L of cell dry weight.

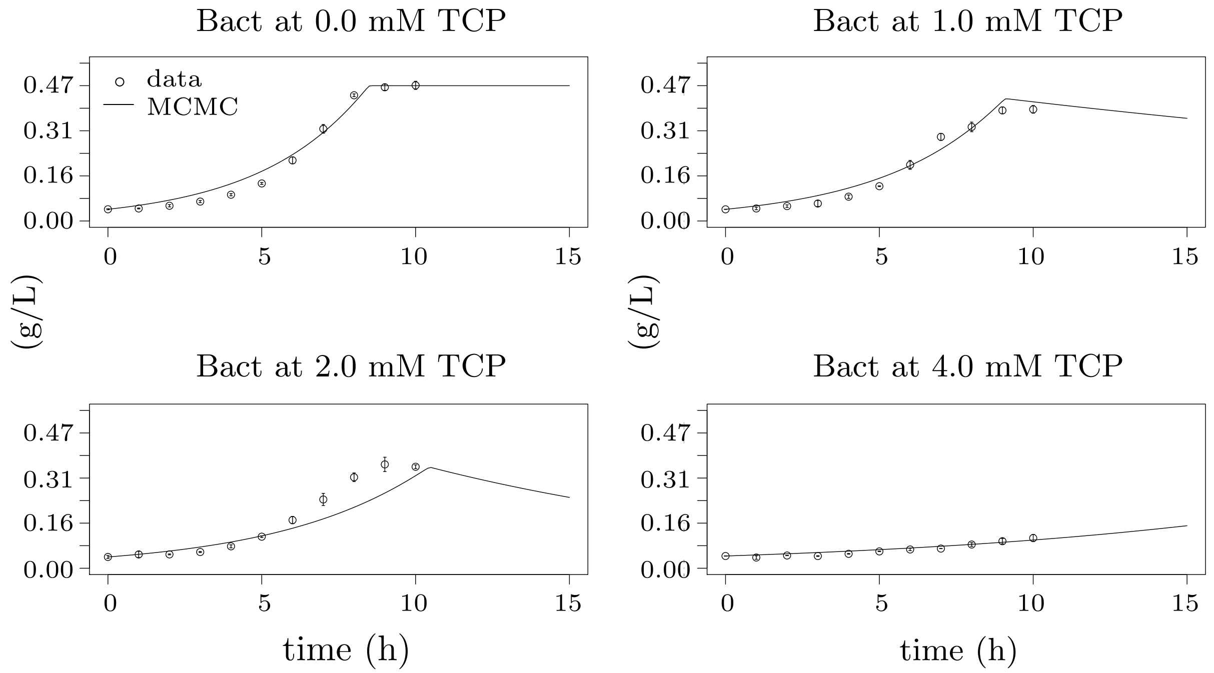


Figure S9: **The results of fitting to population growth data reflecting toxicity caused by TCP — prolonged simulation.**
The plot contains four figures, each showing fitting of the same model to the bacterial population growth data for different concentration of the pathway substrate (TCP) for 15 hours. Although the experimental data are available only for the first 10 hours, the prolonged simulation clearly shows that the model we decided to use fits the data in a good way. The experimental data are pictured as points with standard error bars, the solid lines show the final results of fitting optimised by MCMC method of the FME package which show the best agreement with the experimental data. The model with the best fit appears to be a combination of Monod function (describing the traditional bacterial growth) and Moser function (describing the degradation of the population caused by TCP presence). The x-axes show the time of experiment in hours while the y-axis of the plots show the bacterial population in g/L of cell dry weight.

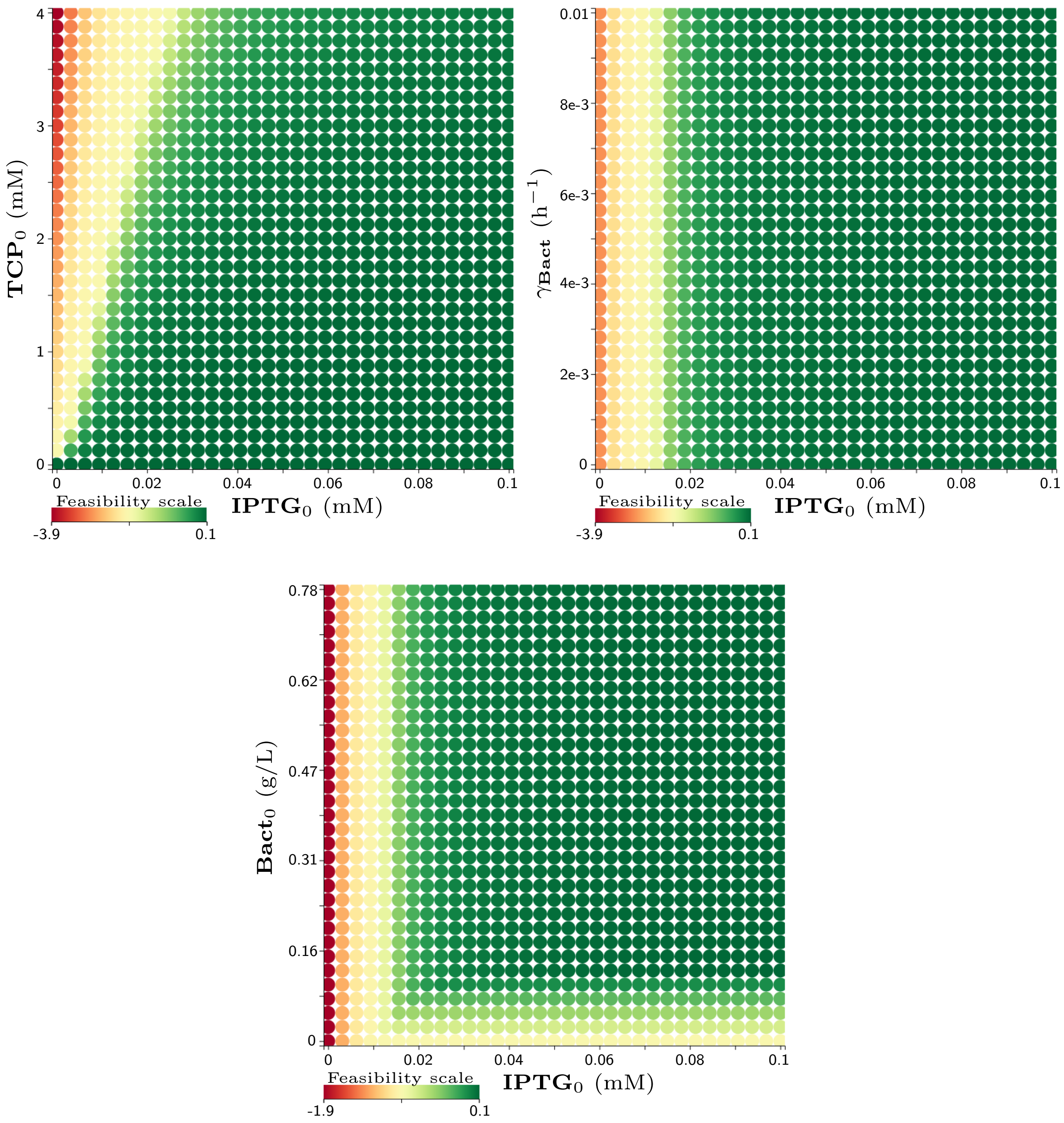


Figure S10: **The results of robustness monitoring for Property 6.**
The figure contains 2-dimensional plots with various coloured circles pointing by their centre to the particular setting of the plotted parameters (or variables) in the particular plot. Initial values of variables and considered parameters (if not displayed in any axis) are: $\text{Bact}_0 = 0.487$ (g/L); $\text{GLY}_0$, $(R)$-$\text{DCP}_0$, $(S)$-$\text{DCP}_0$, $\text{ECH}_0$, $\text{CPD}_0$, $\text{GDL}_0$, $\text{TCP}_0 = 0$ (mM); $\gamma_{\text{Bact}} = 0.0022$ ($\text{h}^{-1}$). All the constants can be found in Figure 11. The shades of green colour imply a feasibility of the particular property in the particular initial setting while the shades of red imply a violation of the property — darker the tone stronger the feasibility/violation. At the bottom of the plots, there are the feasibility scales mapped to real values. All plots represent a single layer of the entire parameter space.

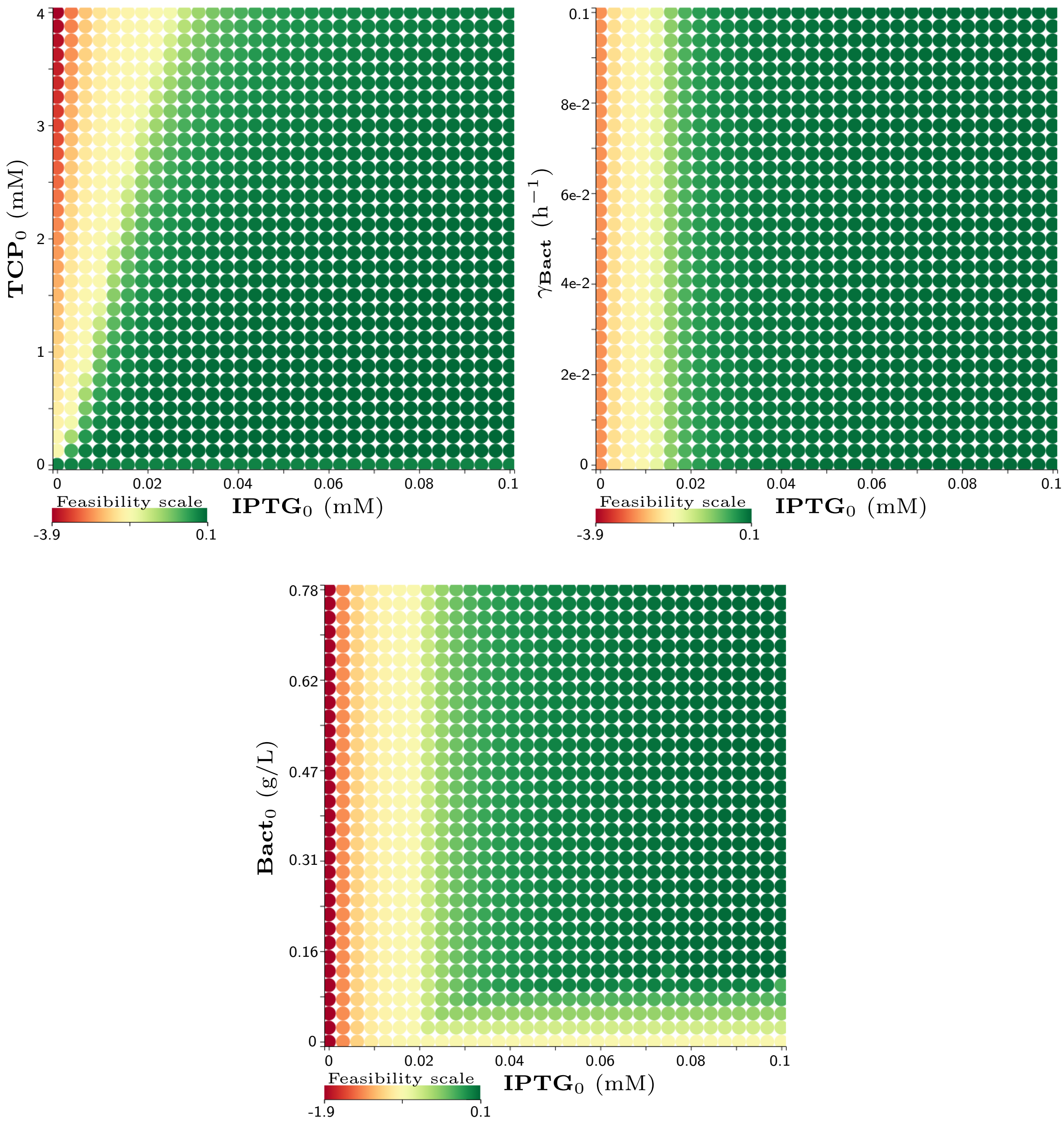


Figure S11: **The result of robustness monitoring for Property 6 with extended range of the death rate coefficient.**
The figure contains 2-dimensional plots with various coloured circles pointing by their centre to the particular setting of the plotted parameters (or variables) in the particular plot. Here, the range of $\gamma_{\text{Bact}}$ is extended up to 0.1 (h$^{-1}$). Initial values of variables and considered parameters (if not displayed in any axis) are: Bact$_0$ = 0.487 (g/L); GLY$_0$, ($R$)-DCP$_0$, ($S$)-DCP$_0$, ECH$_0$, CPD$_0$, GDL$_0$, TCP$_0$ = 0 (mM); $\gamma_{\text{Bact}}$ = 0.1 (h$^{-1}$). All the constants can be found in Figure 11. The shades of green colour imply a feasibility of the particular property in the particular initial setting while the shades of red imply a violation of the property — darker the tone stronger the feasibility/violation.There are the feasibility scales mapped to real values. Each plot represents a single layer of the entire parameter space.

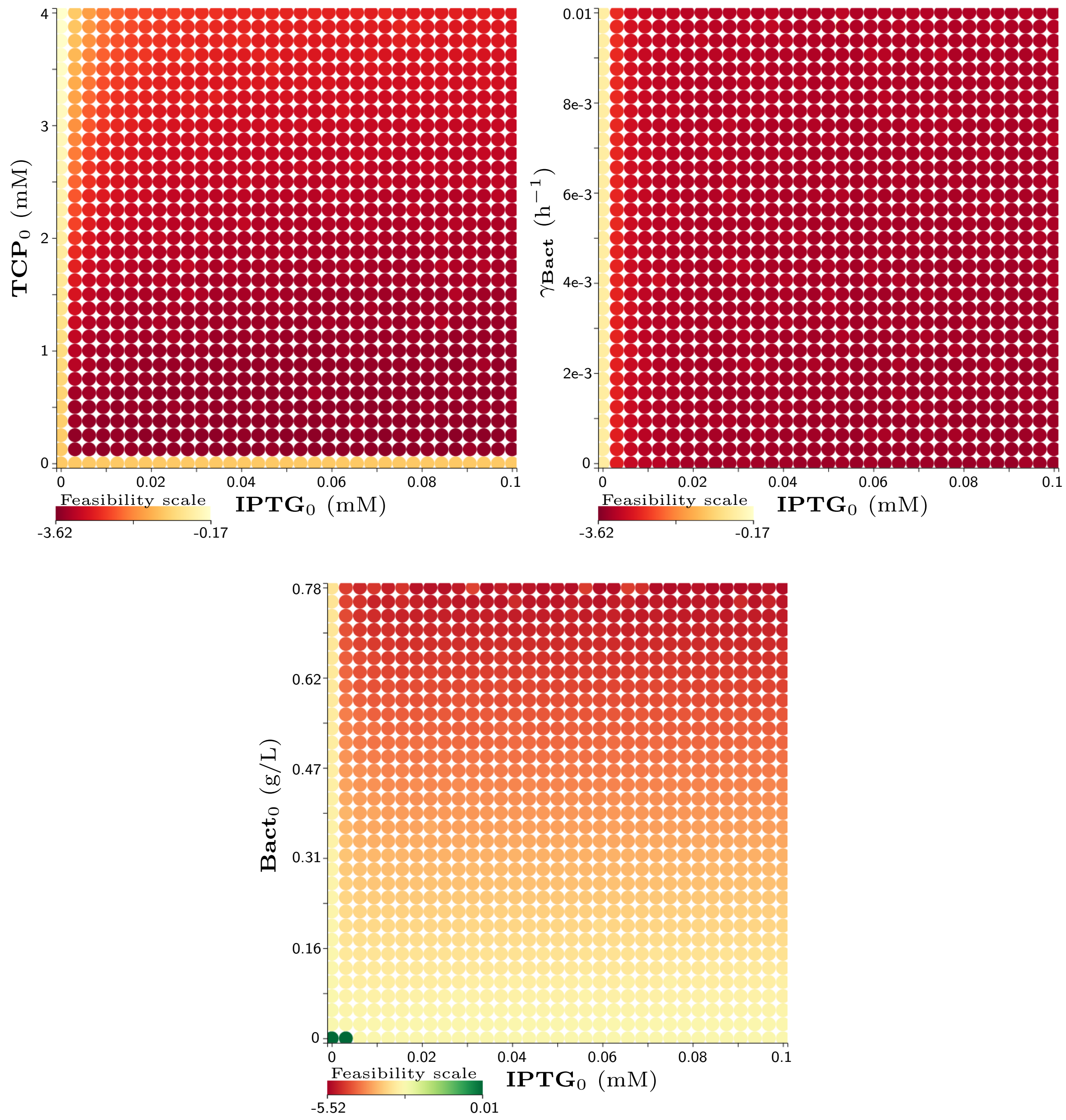


Figure S12: **The results of robustness monitoring for Property 7.**
The figure shows 2D plots with various coloured circles pointing by their centre to the particular initial setting. Initial values of variables and considered parameters (if not displayed in any axis) are: $\text{Bact}_0 = 0.487$ (g/L); $\text{GLY}_0$, $(R)$-$\text{DCP}_0$, $(S)$-$\text{DCP}_0$, $\text{ECH}_0$, $\text{CPD}_0$, $\text{GDL}_0$, $\text{TCP}_0 = 0$ (mM); $\gamma_{\text{Bact}} = 0.0022$ ($\text{h}^{-1}$). All the constants can be found in Figure 11. The shades of green colour imply a feasibility of the property in the particular initial setting while the shades of red imply a violation of the property — darker the tone stronger the feasibility/violation. There are the feasibility scales mapped to real values. Each plot represents a single layer of the entire parameter space. The border conditions where one of shown parameters (or variables) have zero value do not give relevant results because these conditions contradict the basis of the property.

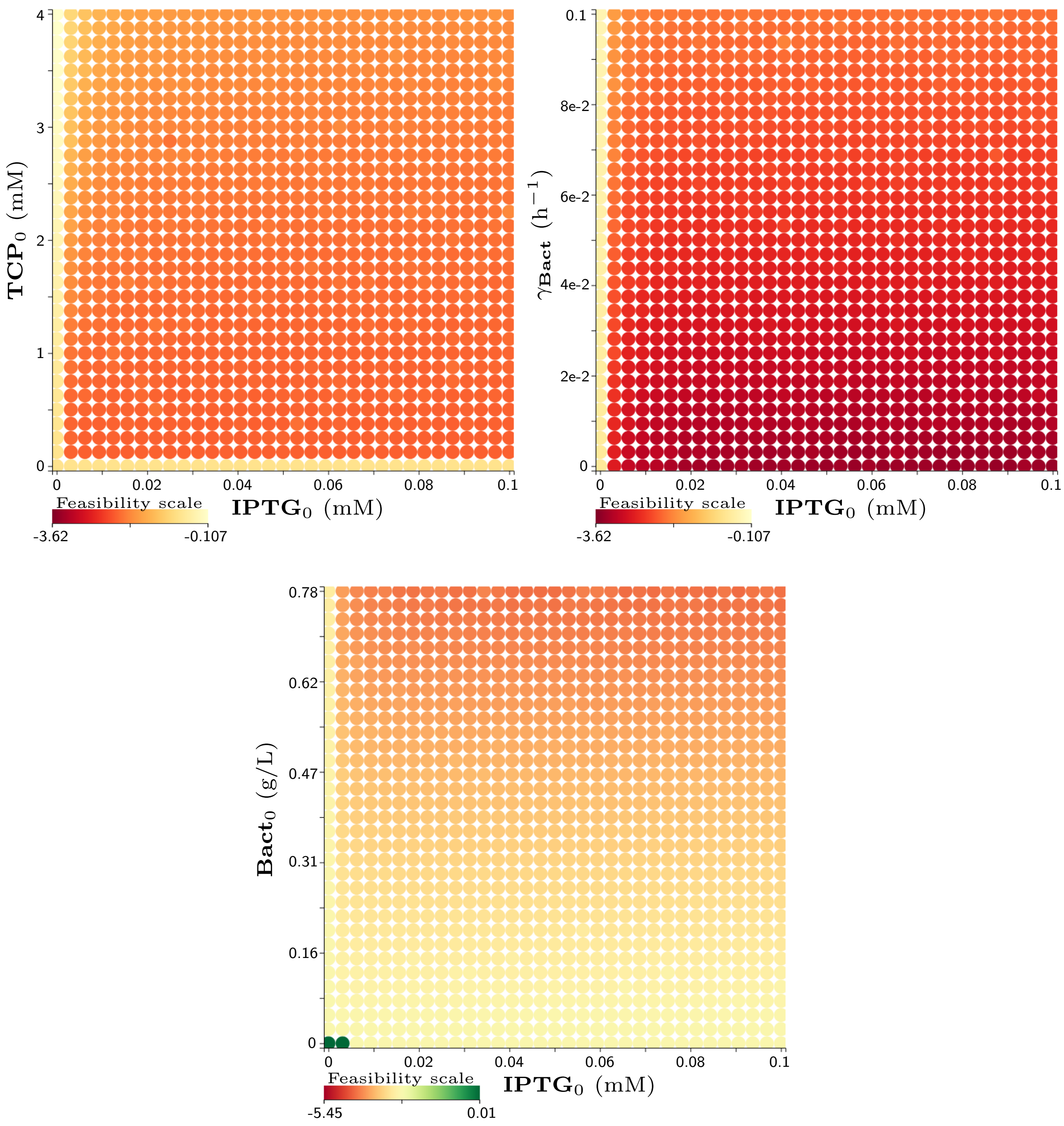


Figure S13: **The results of robustness monitoring for Property 7 with extended range of the death rate coefficient.**

The figure shows 2D plots with various coloured circles pointing by their centre to the particular initial setting. Here, the range of $\gamma_{\mathrm{Bact}}$ is extended up to 0.1 ($h^{-1}$). Initial values of variables and considered parameters (if not displayed in any axis) are: $\mathrm{Bact}_0 = 0.487$ (g/L); $\mathrm{GLY}_0$, ($R$)-$\mathrm{DCP}_0$, ($S$)-$\mathrm{DCP}_0$, $\mathrm{ECH}_0$, $\mathrm{CPD}_0$, $\mathrm{GDL}_0$, $\mathrm{TCP}_0 = 0$ (mM); $\gamma_{\mathrm{Bact}} = 0.1$ ($h^{-1}$). All the constants can be found in Figure 11. The shades of green colour imply a feasibility of the particular property in the particular initial setting while the shades of red imply a violation of the property — darker the tone stronger the feasibility/violation.There are the feasibility scales mapped to real values. Each plot represents a single layer of the entire parameter space. The border conditions where one of shown parameters (or variables) have zero value do not give relevant results because these conditions contradict the basis of the property.
